# Artificial microRNAs and synthetic *trans*‐acting small interfering RNAs interfere with viroid infection

**DOI:** 10.1111/mpp.12529

**Published:** 2017-03-09

**Authors:** Alberto Carbonell, José‐Antonio Daròs

**Affiliations:** ^1^ Instituto de Biología Molecular y Celular de Plantas (Consejo Superior de Investigaciones Científicas‐Universidad Politécnica de Valencia) Valencia 46022 Spain

**Keywords:** artificial microRNA, pathogen resistance, PSTVd, RNA silencing, small RNA, synthetic trans‐acting siRNA, viroid

## Abstract

Artificial microRNAs (amiRNAs) and synthetic *trans*‐acting small interfering RNAs (syn‐tasiRNAs) are two classes of artificial small RNAs (sRNAs) engineered to silence endogenous transcripts as well as viral RNAs in plants. Here, we explore the possibility of using amiRNAs and syn‐tasiRNAs to specifically interfere with infections by viroids, small (250–400‐nucleotide) non‐coding circular RNAs with compact secondary structure infecting a wide range of plant species. The combined use of recent high‐throughput methods for artificial sRNA construct generation and the *Potato spindle tuber viroid* (PSTVd)–*Nicotiana benthamiana* pathosystem allowed for the simple and time‐effective screening of multiple artificial sRNAs targeting sites distributed along PSTVd RNAs of (+) or (–) polarity. The majority of amiRNAs were highly active in agroinfiltrated leaves when co‐expressed with an infectious PSTVd transcript, as were syn‐tasiRNAs derived from a construct including the five most effective amiRNA sequences. A comparative analysis showed that the effects of the most effective amiRNA and of the syn‐tasiRNAs were similar in agroinfiltrated leaves, as well as in upper non‐agroinfiltrated leaves in which PSTVd accumulation was significantly delayed. These results suggest that amiRNAs and syn‐tasiRNAs can be used effectively to control viroid infections in plants.

Artificial microRNAs (amiRNAs) and synthetic *trans*‐acting small interfering RNAs (syn‐tasiRNAs) are two classes of artificial small RNAs (sRNAs) engineered to silence specific transcripts in plants. They are produced *in planta* by the expression of a functional miRNA or tasiRNA precursor with modified miRNA/miRNA* or tasiRNA sequences, respectively. amiRNAs and syn‐tasiRNAs are functionally similar, but are generated differently. Although amiRNAs derive from DICER‐LIKE1 (DCL1) cleavage of miRNA precursors with foldback structures, syn‐tasiRNAs are produced in a multi‐step process including: (i) cleavage of a *TAS* precursor by an miRNA/ARGONAUTE (AGO) complex, (ii) conversion of one of the cleavage products to double‐stranded RNA (dsRNA) by RNA‐DEPENDENT RNA POLYMERASE 6, and (iii) sequential processing by DCL4 of the dsRNA into 21‐nucleotide phased syn‐tasiRNAs in register with the miRNA‐guided cleavage site. One of the strands of the amiRNA or syn‐tasiRNA duplex associates with an AGO protein to silence highly sequence complementary transcripts, usually through AGO‐mediated endonucleolytic cleavage (Tiwari *et al*., 2014; Zhang, 2014).

Both classes of artificial sRNAs have been used to selectively inactivate endogenous and reporter genes (Alvarez *et al*., 2006; de la Luz Gutierrez‐Nava *et al*., 2008; Schwab *et al*., 2006), as well as viral RNAs to confer antiviral resistance in transgenic plants (Niu *et al*., 2006; Singh *et al*., 2015). Recently, a high‐throughput platform for the time‐ and cost‐effective design and synthesis of amiRNAs and syn‐tasiRNAs has been reported and includes: (i) the *P‐SAMS* tool (http://p-sams.carringtonlab.org) for the automated design of specific plant amiRNAs and syn‐tasiRNAs (Fahlgren *et al*., 2016), and (ii) the generation of amiRNA and syn‐tasiRNA constructs by direct ligation of annealed DNA oligonucleotides containing the desired amiRNA or syn‐tasiRNA(s) sequences into a new generation of plant ‘B/c’ expression vectors (Carbonell *et al*., 2014, 2015).

Viroids are plant‐specific infectious agents that affect a wide range of crop and ornamental species. They are composed of a small (250–400‐nucleotide), circular, single‐stranded RNA, with a compact secondary structure and without protein‐coding capacity (Daros, 2016; Flores *et al*., 2015). Viroids replicate through an RNA‐based rolling‐circle mechanism (Branch and Robertson, 1984) with the plausible involvement of dsRNA intermediates and the presence of RNAs of (+) and (–) polarities. They are classified into the families *Pospiviroidae* or *Avsunviroidae*, whose members replicate and accumulate in the nucleus and the chloroplast of plant cells, respectively. *Potato spindle tuber viroid* (PSTVd), the type species of the family *Pospiviroidae* (Diener, 1972; Gross *et al*., 1978), naturally infects economically important crops, such as potato (*Solanum tuberosum*) and tomato (*Solanum lycopersicum*). PSTVd is currently considered to be an important threat to plant health and is classified as a quarantine pathogen in certain regions of the world (Tsuda and Sano, 2014).

Like viruses, viroids are inducers and targets of the plant RNA silencing machinery, as inferred by the accumulation of viroid‐derived siRNAs (vd‐siRNAs) during infections of viroids of both families (Itaya *et al*., 2001; Martinez de Alba *et al*., 2002; Papaefthimiou *et al*., 2001). These results implicate DCLs in vd‐siRNA biogenesis, presumably from their action on the dsRNA replicative intermediates or on the genomic structured RNAs. Recently, the observation that specific AGOs selectively bind viroid‐derived sRNAs (vd‐sRNAs) and attenuate viroid accumulation *in vivo* suggests that viroid RNAs could also be targeted by AGO/vd‐sRNAs complexes (Minoia *et al*., 2014), as proposed for viruses (Carbonell and Carrington, 2015).

RNA‐based resistance to viroids (reviewed in Dalakouras *et al*., 2015) was initially achieved through the transgenic expression of antisense RNAs (Matousek *et al*., 1994), hammerhead ribozymes (Atkins *et al*., 1995; Carbonell *et al*., 2011; Yang *et al*., 1997) or dsRNA ribonucleases (Sano *et al*., 1997). Later, RNA silencing‐based strategies showed that antiviroid resistance could be obtained through the co‐inoculation of large amounts of dsRNA molecules of viroid sequence (Carbonell *et al*., 2008), or through the expression of constructs producing hairpin RNAs of viroid sequence (Adkar‐Purushothama *et al*., 2015; Carbonell *et al*., 2008; Schwind *et al*., 2009). Despite their effectiveness, these potent approaches have major limitations, such as the lack of high specificity or the tediousness of the generation of hairpin constructs or of large amounts of dsRNAs. The use of more recent and specific tools, such as amiRNAs or syn‐tasiRNAs, has not yet been reported for the engineering of antiviroid resistance.

In this work, the goal was to explore whether amiRNAs and syn‐tasiRNAs could be used to interfere specifically with viroid infections. We combined the use of recently developed high‐throughput methods for plant artificial sRNA construct generation (Carbonell *et al*., 2014, 2015; Fahlgren *et al*., 2016) with the PSTVd–*Nicotiana benthamiana* pathosystem to confirm, in a simple and time‐effective manner, that both classes of artificial sRNAs can inhibit viroid infection. The main steps followed are summarized in a workflow diagram in Fig. S1 (see Supporting Information).

*P‐SAMS* (Fahlgren *et al*., 2016) was used to generate a list of 162 and 149 amiRNAs targeting specifically (+) or (–) RNAs, respectively, of the RG1 strain (GenBank Acc. No. U23058) of PSTVd (Gruner *et al*., 1995) with no predicted off‐targets in *S. lycopersicum* (Methods S1, Tables S1 and S2, Text S1, see Supporting Information). PSTVd‐RG1 causes severe stunting and leaf curling symptoms in tomato, but is asymptomatic in *N. benthamiana* (Matousek *et al*., 2007). Six amiRNAs targeting PSTVd(+) RNAs and six amiRNAs targeting PSTVd(–) RNAs were selected based on the following criteria: (i) amiRNAs extensively base pair with target RNA; (ii) target sites are distributed along the five structural domains of PSTVd (Keese and Symons, 1985), and are included in both sequence‐conserved (C domain) and variable regions (other domains); and (iii) cleavage sites are in internal loops, or in bulged or stem regions (Figs 1A and 2A). In addition, two of 633 (Table S3, see Supporting Information) amiRNAs targeting *Escherichia coli* β‐glucuronidase (GUS) transcripts were selected as controls based on similar criteria (Fig. S2, see Supporting Information). *TargetFinder* (Fahlgren and Carrington, 2010) was used to confirm that all selected amiRNAs had no significant off‐targets in *N. benthamiana* (Table S4, see Supporting Information). Selected amiRNA sequences (Figs 1B, 2B and S2, Text S2, see Supporting Information) were transferred to *pMDC32B‐AtMIR390a‐B/c* vector (Methods S1, Table S5, see Supporting Information), previously validated for efficient, one‐step cloning of amiRNA inserts and high expression of amiRNAs in different plant species, including *N. benthamiana* (Carbonell *et al*., 2014). Anti‐PSTVd amiRNAs accumulated to different levels in *N. benthamiana* agroinfiltrated leaves as shown by Northern blot analysis (Fig. S3, see Supporting Information).

**Figure 1:**
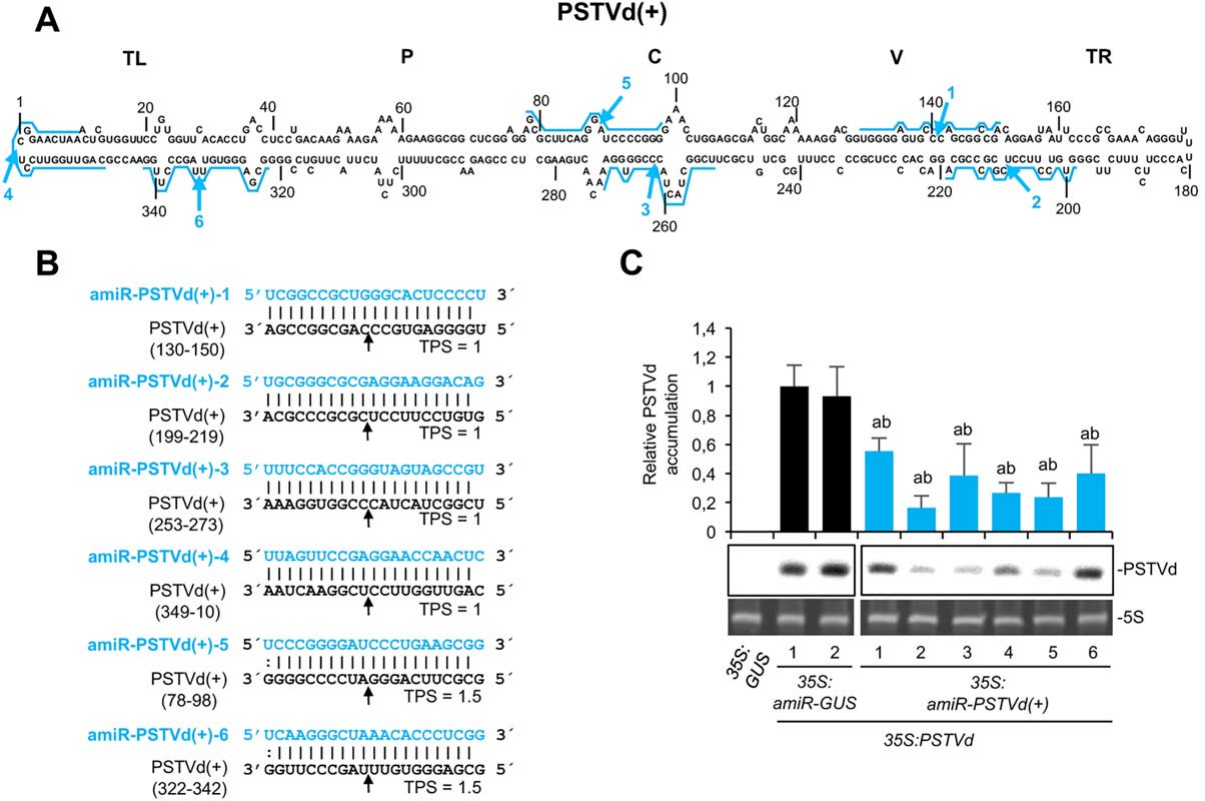
Analysis of the antiviroid activity of several artificial microRNAs (amiRNAs) targeting *Potato spindle tuber viroid* (PSTVd)(+) RNAs. (A) Diagram of the rod‐like secondary structure of the minimum free energy of PSTVd genomic RNA of (+) polarity predicted computationally by *RNAfold*. amiRNA cleavage sites in PSTVd(+) RNAs are indicated by blue arrows, and complete amiRNA target sites are highlighted with blue lines. The five structural domains in the members of the family *Pospiviroidae* are indicated: TL, terminal left; P, pathogenicity; C, central conserved; V, variable; TR, right terminal. (B) Base pairing of amiRNAs and target PSTVd(+) RNAs. Anti‐PSTVd(+) amiRNA and PSTVd(+) sequences are shown in blue and black, respectively. Coordinates of the complete target site in PSTVd(+) RNAs are given. The TPS (or ‘Target Prediction Score’) value is output by P‐SAMS and accounts for the degree of base pairing between amiRNA and target (‘1’ being the value of maximal base pairing). The black arrows indicate the amiRNA‐predicted cleavage site. (C) Northern blot detection of PSTVd(+) genomic RNA (circular forms) in preparations from *Nicotiana benthamiana* agroinfiltrated leaves at 2 days post‐agroinfiltration. A representative blot corresponding to one of the three biological replicates is shown. Each biological replicate is a pool of two agroinfiltrated leaves from the same plant. 5S rRNA stained with ethidium bromide was used as loading control. The graph at the top shows the mean (*n* = 3) + standard deviation (SD) circular PSTVd(+) accumulation relative to that of a control sample (*35S:amiR‐GUS‐1* + *35S:PSTVd*). Bars with the letters ‘a’ and ‘b’ are statistically significantly different from that of sample *35S:amiR‐GUS‐1 + 35S:PSTVd* and *35S:amiR‐GUS‐2 + 35S:PSTVd*, respectively (*P* < 0.05 for all pair‐wise Student's *t*‐test comparisons). The experiment was repeated with similar results.

**Figure 2:**
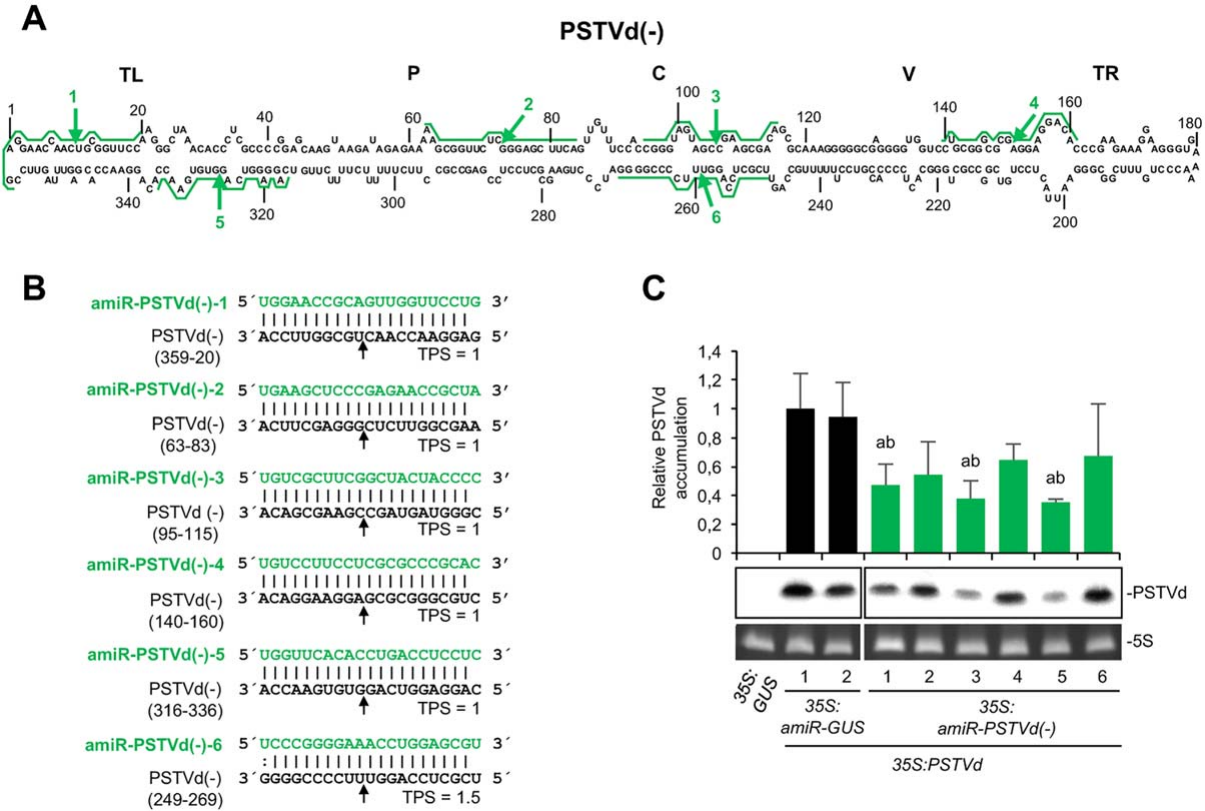
Analysis of the antiviroid activity of several artificial microRNAs (amiRNAs) targeting *Potato spindle tuber viroid* (PSTVd)(–) RNAs. (A) Diagram of the secondary structure of the minimum free energy of linear PSTVd monomeric RNA of (–) polarity predicted computationally by *RNAfold*. It should be noted that PSTVd RNAs of (–) polarity exist *in vivo* as part of long multimeric oligomers. amiRNA cleavage sites in PSTVd(–) RNAs are indicated by green arrows and complete amiRNA target sites are highlighted with green lines. Other details are the same as in Fig. 1A. (B) Base pairing of amiRNAs and target PSTVd(–) RNAs. Anti‐PSTVd(–) amiRNA and PSTVd(–) sequences are shown in green and black, respectively. Coordinates of the complete target site in PSTVd(–) RNAs are given. Other details are the same as in Fig. 1B. (C) Northern blot detection of PSTVd(+) genomic RNAs (circular forms) in preparations from *Nicotiana benthamiana* agroinfiltrated leaves at 2 days post‐agroinfiltration. Other details are the same as in Fig. 1C.

To determine the antiviroid activity of selected amiRNAs, PSTVd accumulation was analysed in *N. benthamiana* leaves co‐agroinfiltrated independently with each of the amiRNA constructs together with the *35S:PSTVd* construct. *35S:PSTVd* expresses a highly infectious dimeric PSTVd(+) transcript (Methods S1). Agroinfiltrated leaves were harvested at 2 days post‐agroinfiltration (dpa), and RNAs were purified and analysed by denaturing polyacrylamide gel electrophoresis (PAGE) followed by Northern blot hybridization, to detect genomic circular PSTVd(+) RNAs, the progeny of viroid infection. In a first experiment, PSTVd accumulated to significantly lower levels in all samples including anti‐PSTVd(+) amiRNAs compared with samples including anti‐GUS amiRNAs (*P* < 0.05 for all pair‐wise Student's *t*‐test comparisons; Fig. 1C). In a second experiment, PSTVd accumulation was also reduced in all samples containing anti‐PSTVd(–) amiRNAs compared with control samples, although, in this case, the decrease was statistically significant only in one‐half of the samples including anti‐PSTVd amiRNAs [*P* < 0.05 for pair‐wise Student's *t*‐test comparisons including amiR‐PSTVd(–)‐1, amiR‐PSTVd(–)‐3 and amiR‐PSTVd(–)‐5; Fig. 2C]. PSTVd progenies from all samples including anti‐PSTVd amiRNAs were sequenced and, in all cases, target sites did not present mutations. Taken together, these results indicate that the majority of anti‐PSTVd amiRNAs tested are active, although with different efficacies. In our experimental conditions, amiRNA efficacy cannot be explained exclusively by the amiRNA expression level (Fig. S3). Thus, it is likely that other factors, such as target site accessibility, also contribute to define the efficacy of each amiRNA. Interestingly, despite the fact that, during a viroid infection, viroid RNAs of (+) polarity are in excess compared with those of (–) polarity, it appears that anti‐PSTVd(+) amiRNAs are, in general, more active than anti‐PSTVd(–) amiRNAs. A possible explanation is that anti‐PSTVd(–) amiRNAs may only target oligomeric replication intermediates of (–) polarity, whereas anti‐PSTVd(+) amiRNAs might target not only replication intermediates of (+) polarity, but also primary dimeric PSTVd(+) transcripts and genomic circular PSTVd(+) RNAs. Indeed, it seems that the majority of the genomic PSTVd(+) RNAs might be accessible to amiRNAs, as suggested by the recent verification by *in vivo* selective 2′‐hydroxyl acylation analysed by primer extension (SHAPE) that PSTVd(+) RNA accumulates in *N. benthamiana* as a ‘naked’ molecule rather than tightly associated with host proteins (Lopez‐Carrasco and Flores, 2016). Interestingly, amiR‐PSTVd(–)‐5, the most effective anti‐PSTVd(–) amiRNA, targets the same genomic region as an efficient anti‐PSTVd(–) hammerhead ribozyme overexpressed in *N. benthamiana* and *S. tuberosum* (Carbonell *et al*., 2011; Yang *et al*., 1997), suggesting that this region could be particularly accessible. Finally, although the three more effective anti‐PSTVd(+) amiRNAs target bulged regions, the three more effective anti‐PSTVd(–) amiRNAs target stems; thus, no correlation can be made between amiRNA efficacy and the secondary structure of its target site.

Next, we generated the *35S:syn‐tasiR‐PSTVd* construct by introducing five syn‐tasiRNA sequences corresponding to the five most effective anti‐PSTVd amiRNA sequences in the *pMDC32B‐AtTAS1c‐B/c* vector (Fig. 3A, Methods S1), previously validated for efficient, one‐step cloning of syn‐tasiRNA inserts and high expression of *TAS1c*‐based syn‐tasiRNAs in different plant species, including *N. benthamiana* (Carbonell *et al*., 2014). Similarly, five syn‐tasiRNA sequences corresponding to amiR‐GUS‐1 and amiR‐GUS‐2 were introduced in *pMDC32B‐AtTAS1c‐B/c* to generate the *35S:syn‐tasiR‐GUS* control (Fig. 3A, Methods S1). The anti‐PSTVd activity of both syn‐tasiRNA constructs was analysed in *N. benthamiana* by co‐agroinfiltration of each syn‐tasiRNA construct together with the *35S:PSTVd* construct and the *35S:MIR173a* construct. miR173a is not present in *N. benthamiana* and thus had to be expressed as *TAS1c*‐dependent tasiRNA biogenesis is triggered by AGO1/miR173a‐mediated cleavage of *TAS1c* transcripts (Montgomery *et al*., 2008). As an additional control, *35S:syn‐tasiR‐PSTVd* was co‐agroinfiltrated with *35S:PSTVd* in the absence of *35S:MIR173a*. Northern blot analysis at 2 dpa of RNA preparations from agroinfiltrated leaves expressing the *35S:PSTVd* construct showed that PSTVd accumulated to significantly lower levels in samples including *35S:syn‐tasiR‐PSTVd* and *35S:MIR173a* compared with control samples including *35S:syn‐tasiR‐GUS* and *35S:MIR173a* (Fig. 3B, lanes 3 and 5, respectively; *P* < 0.03 in Student's *t*‐test comparison) or *35S:syn‐tasiR‐PSTVd* only (Fig. 3B, lanes 4 and 5, respectively; *P* < 0.01 in Student's *t*‐test comparison). Again, PSTVd progenies from samples including anti‐PSTVd syn‐tasiRNAs were sequenced and, in all cases, target sites did not contain mutations. Altogether, these results suggest that PSTVd‐specific syn‐tasiRNAs are active in agroinoculated tissue, and that their activity is dependent on the presence of miR173a.

**Figure 3:**
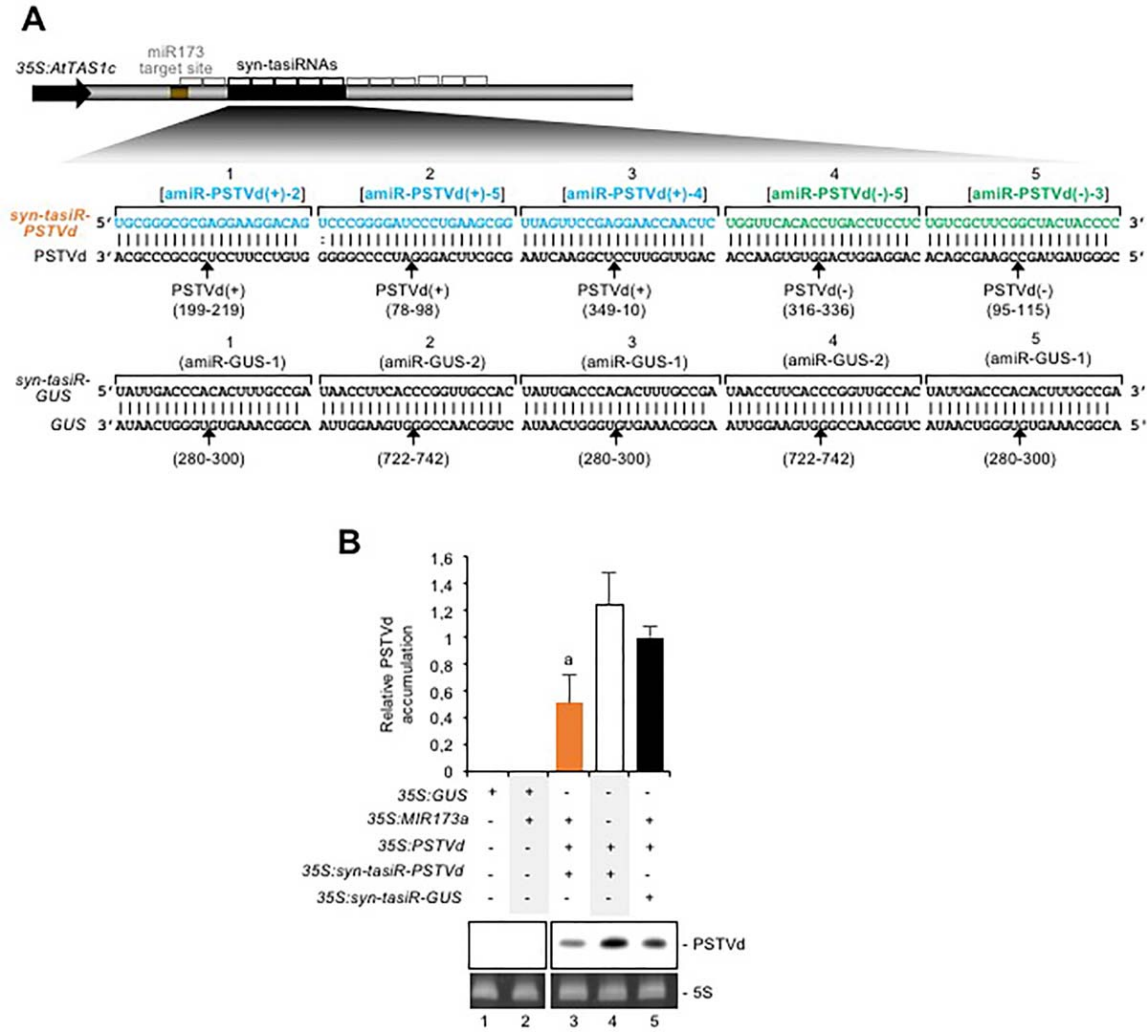
Analysis of the anti‐*Potato spindle tuber viroid* (PSTVd) activity of synthetic *trans*‐acting small interfering RNAs (syn‐tasiRNAs). (A) Organization of syn‐tasiRNA constructs. tasiRNA positions 3′D1[+], 3′D2[+] and 3′D5[+]‐3′D10[+] are indicated by grey brackets, with syn‐tasiRNA positions indicated by black brackets and highlighted in black. Other details are the same as in Figs 1A, 1B and 2B. (B) Northern blot detection of PSTVd(+) genomic RNAs (circular forms) in preparations from *Nicotiana benthamiana* agroinfiltrated leaves at 2 days post‐agroinfiltration. The graph at the top shows the mean (*n* = 3) + standard deviation (SD) PSTVd accumulation relative to that of the control sample (*35S:syn‐tasiR‐GUS + 35S:MIR173a + 35S:PSTVd*). The bar with a letter ‘a’ is statistically significantly different from the bar of the control sample (*P* < 0.03 for all pair‐wise Student's *t*‐test comparisons). Other details are the same as in Fig. 1C.

Next, we compared the inhibitory activity of amiRNAs and syn‐tasiRNAs in both agroinfiltrated and upper non‐agroinfiltrated tissues. To better mimic the situation likely to occur in plants overexpressing artificial sRNAs and challenged with PSTVd (where artificial sRNAs are in excess compared with viroid molecules at the initial stages of the infection), it was first necessary to determine whether the amount of agroinfiltrated culture including *35S:PSTVd* could be lowered without compromising either the infectivity of the construct or the detection of PSTVd RNAs. Hence, we set up a preliminary experiment in which *Agrobacterium tumefaciens* cultures transformed with *35S:PSTVd* were co‐agroinfiltrated at final optical densities (ODs) of 0.5, 0.1 or 0.01 together with the *35S:amiR‐GUS‐1* control. Northern blot analysis of RNA preparations from agroinfiltrated leaves at 2, 3, 4 and 6 dpa showed that, as suspected, PSTVd accumulation increased with time, as well as with the OD of the *A. tumefaciens* culture transformed with *35S:PSTVd* (Fig. S4, see Supporting Information). Because PSTVd accumulation was similar in samples collected at 2 dpa and co‐agroinfiltrated with the *35S:PSTVd* culture at an OD of 0.5 (conditions of experiments from Fig. 2) (Fig. S4, lanes 2 and 10, respectively) compared with samples collected at 4 dpa and co‐agroinfiltrated with the *35S:PSTVd* culture at the lowest OD of 0.01, the latter conditions were used in subsequent analyses.

To simplify the analyses, we studied the antiviroid effects of *35S:amiR‐PSTVd(+)‐2*, the most effective anti‐PSTVd amiRNA construct, and *35S:syn‐tasiR‐PSTVd* compared with *35S:GUS*, when co‐agroinfiltrated with *35S:PSTVd*. Because *35S:MIR173a* had to be co‐expressed with *35S:syn‐tasiR‐PSTVd* to trigger syn‐tasiRNA biogenesis, it was also co‐expressed in samples including *35S:amiR‐PSTVd(+)‐2* or *35S:GUS* for comparative purposes. In a first experiment, Northern blot analysis at 4 dpa of RNA preparations from agroinfiltrated leaves showed that PSTVd accumulated to significantly lower levels in samples including *35S:PSTVd, 35S:MIR173a* and *35S:amiR‐PSTVd(+)‐2* or *35S:syn‐tasiR‐PSTVd* compared with control samples including *35S:GUS*, *35S:PSTVd* and *35S:MIR173a* (*P* < 0.01 for all pair‐wise Student's *t*‐test comparisons; Fig. 4A, lanes 3, 4 and 1, respectively). In particular, PSTVd accumulation was slightly (but not significantly) reduced in samples including *35S:amiR‐PSTVd(+)‐2* compared with samples including *35S:syn‐tasiR‐PSTVd* (*P* = 0.101 in Student's *t*‐test comparison; Fig. 4A, lanes 3 and 4, respectively). Target sites with the wild‐type sequence were recovered when sequencing PSTVd progenies from samples including anti‐PSTVd artificial sRNAs.

**Figure 4:**
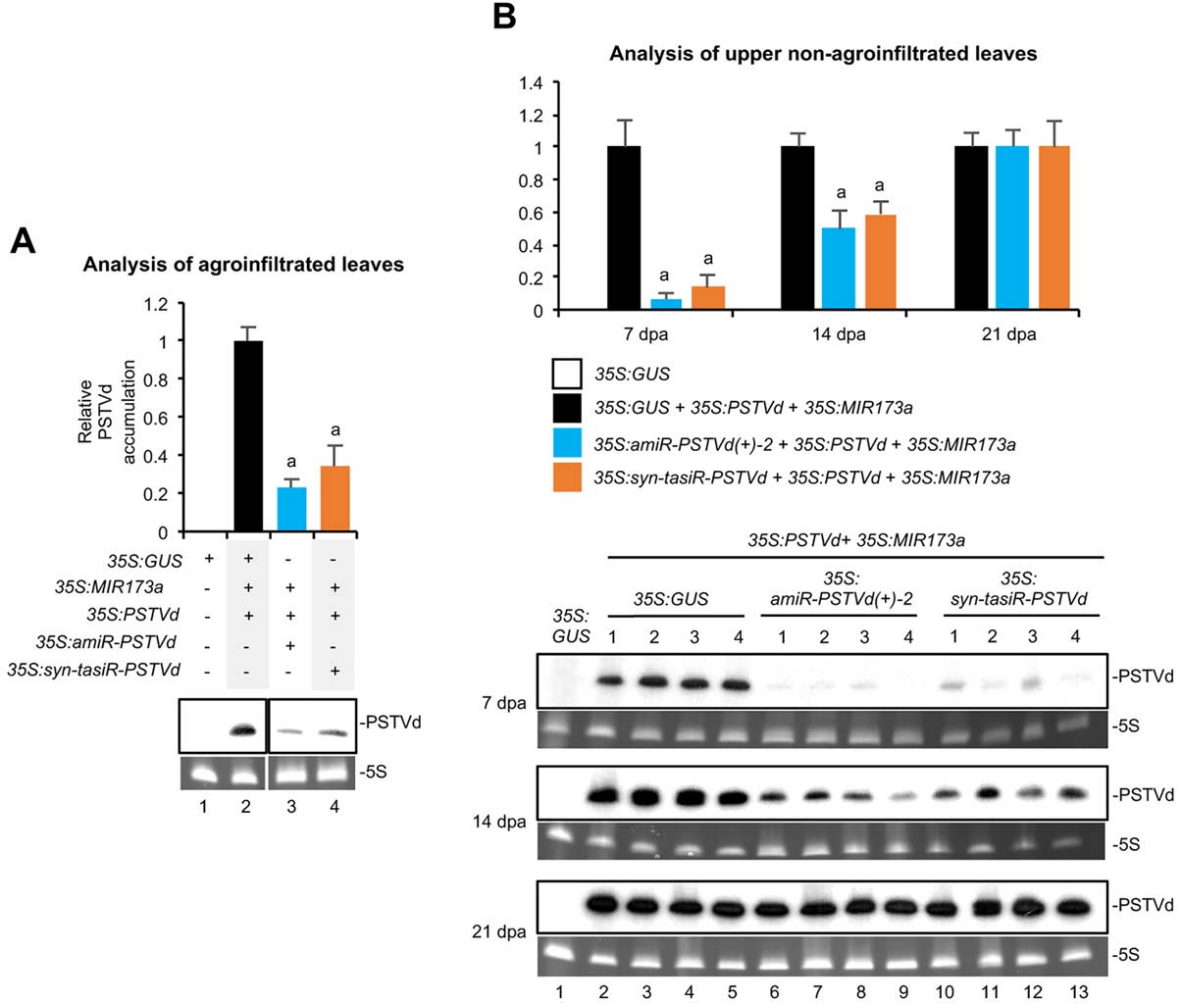
Comparative analysis of the anti‐*Potato spindle tuber viroid* (PSTVd) activity of artificial microRNAs (amiRNAs) and synthetic *trans*‐acting small interfering RNAs (syn‐tasiRNAs). (A) Northern blot detection of PSTVd(+) genomic RNA (circular forms) in preparations from *Nicotiana benthamiana* agroinfiltrated leaves at 4 days post‐agroinfiltration (dpa). The graph at the top shows the mean (*n* = 3) + standard deviation (SD) PSTVd accumulation relative to that of the control sample (*35S:GUS + 35S:MIR173a + 35S:PSTVd*). Bars with letters are statistically significantly different from that of the control sample (*P* < 0.01 for all pair‐wise Student's *t*‐test comparisons). Other details are the same as in Fig. 1C. (B) Northern blot detection of PSTVd(+) genomic RNA (circular forms) in preparations from *N. benthamiana* upper non‐agroinfiltrated leaves at 7, 14 and 21 dpa. Each biological replicate is a pool of the two youngest, fully expanded leaves of each plant at each time point. Other details are the same in (A) and Fig. 1C.

To study the effects of both classes of artificial sRNAs on viroid systemic infection, a similar experiment was conducted in which RNA preparations from upper non‐agroinfiltrated leaves were analysed at different dpa (Fig. 4B). Northern blot analysis revealed that PSTVd accumulation decreased notably in upper leaves from plants co‐agroinfiltrated with *35S:PSTVd, 35S:MIR173a* and *35S:amiR‐PSTVd(+)‐2* or *35S:syn‐tasiR‐PSTVd* with respect to samples from plants co‐agroinfiltrated with *35S:GUS*, *35S:PSTVd* and *35S:MIR173a* (Fig. 4B, lanes 6–13 compared with lanes 2–5, respectively). The effect was clearly visible in leaves collected at 7 dpa, decreased slightly at 14 dpa and disappeared at 21 dpa. Because amiR‐PSTVd(+)‐2 and syn‐tasiR‐PSTVd were expressed transiently in agroinfiltrated leaves and were not expected to move systemically, it is not surprising that the anti‐PSTVd effects of both artificial sRNAs decreased over time. Sequencing of PSTVd progenies in systemic tissue from plants agroinfiltrated with anti‐PSTVd artificial sRNAs showed that, in all cases, target sites did not contain mutations. In any case, these results confirm that PSTVd RNAs can be down‐regulated with specific amiRNAs and syn‐tasiRNAs. Moreover, in our experimental conditions, both classes of artificial sRNAs produced similar protective effects. Possibly, *35S:amiR‐PSTVd(+)‐2*, *35S:syn‐tasiR‐PSTVd* or any other construct expressing an active anti‐PSTVd amiRNA may induce resistance against PSTVd when stably expressed in transgenic plants. However, we cannot rule out the possibility that, during infections, specific mutations in target sites included in PSTVd RNAs could compromise the resistance, as observed for certain amiRNA target sites in viral RNAs (Carbonell *et al*., 2016; Lafforgue *et al*., 2011). This situation seems unlikely to occur for target sites included in sequence‐conserved regions, such as the central conserved region in the C domain of PSTVd (Keese and Symons, 1985). Thus, we suspect that amiRNAs, such as amiR‐PSTVd(+)‐3 and amiR‐PSTVd(+)‐5, targeting effectively conserved regions of the C domain could induce a durable resistance. In addition, syn‐tasiRNA‐based resistance is expected to be highly durable considering that several target sites would have to mutate simultaneously to break the resistance (Carbonell *et al*., 2016).

Similar protective effects have been described previously in upper non‐agroinfiltrated leaves of *N. benthamiana* plants co‐agroinfiltrated with a similar dimeric PSTVd construct together with a hairpin construct including an inverted repeat comprising the PSTVd upper strand of the rod‐like structure separated by an intron (Carbonell *et al*., 2008). Later, it was shown that the expression in T3 transgenic tomato of a hairpin construct including a truncated PSTVd sequence and a near full‐length PSTVd sequence with minor modifications in sense and antisense orientations, respectively, induced resistance to PSTVd (Schwind *et al*., 2009). Intriguingly, the same hairpin construct expressed in T1 transgenic tomatoes induced viroid‐like symptoms in the absence of PSTVd (Wang *et al*., 2004). The authors suggested that siRNAs derived from the hairpin construct may target endogenous transcripts and induce symptoms. Certainly, this observation highlights one of the main limitations of the hairpin approach, which is the lack of high specificity because of the possible generation of siRNAs that could target sequence complementary endogenous transcripts and lead to undesired off‐target effects. In contrast, the two strategies described here, amiRNA and syn‐tasiRNA, should minimize this type of off‐target effect. Indeed, high amiRNA target specificity has been shown in amiRNA‐expressing transgenic plants, first through genome‐wide expression profiling (Schwab *et al*., 2006) and, more recently, through genome‐wide transcriptome profiling combined with 5′‐RNA ligase‐mediated rapid amplification of cDNA ends (5′‐RLM‐RACE) analysis (Carbonell *et al*., 2015).

In conclusion, we have used recent high‐throughput methods and the PSTVd–*N. benthamiana* pathosystem to evaluate, in a simple and time‐effective manner, the antiviroid activity of multiple artificial sRNA constructs designed to target specifically PSTVd RNAs. Notably, both amiRNAs and syn‐tasiRNAs expressed transiently are similarly effective in interfering specifically with PSTVd infection. We suspect that active amiRNAs or syn‐tasiRNAs targeting conserved regions could induce long‐lasting resistance to PSTVd when expressed stably in transgenic plants. Future work is thus needed to confirm that these promising artificial sRNA‐based strategies can be applied to induce high levels of durable antiviroid resistance in plants.

## Supporting information

Additional Supporting Information may be found in the online version of this article at the publisher's website:

**Fig. S1** Diagram of the steps for the design and synthesis of anti‐*Potato spindle tuber viroid* (PSTVd) artificial microRNAs (amiRNAs) and synthetic *trans*‐acting small interfering RNAs (syn‐tasiRNAs), and for the analysis of their antiviroid activity.

**Fig. S2** Base pairing of artificial microRNAs (amiRNAs) and target β‐glucuronidase (GUS) RNAs.

**Fig. S3** Analysis of the expression of anti‐*Potato spindle tuber viroid* (PSTVd) artificial microRNAs (amiRNAs) in *Nicotiana benthamiana* agroinfiltrated leaves.

**Fig. S4** Analysis of *Potato spindle tuber viroid* (PSTVd) accumulation in *Nicotiana benthamiana* agroinfiltrated leaves at different days post‐agroinfiltration (dpa).

**Methods S1** Experimental procedures.

**Table S1** Summary of *P‐SAMS* optimal results for artificial microRNAs (amiRNAs) targeting *Potato spindle tuber viroid* (PSTVd) (+) RNAs.

**Table S2** Summary of *P‐SAMS* optimal results for artificial microRNAs (amiRNAs) targeting *Potato spindle tuber viroid* (PSTVd) (–) RNAs.

**Table S3** Summary of *P‐SAMS* optimal results for artificial microRNAs (amiRNAs) targeting β‐glucuronidase (GUS).

**Table S4** Summary of *TargetFinder* results for selected artificial microRNAs (amiRNAs) in *Nicotiana benthamiana*.

**Table S5** DNA oligonucleotides used in this study.

**Text S1** DNA sequences in FASTA format of all artificial small RNA‐generating precursors used in this study.

**Text S2** DNA sequences in FASTA format used in P‐SAMS amiRNA Designer to design anti‐*Potato spindle tuber viroid* (PSTVd) or anti‐β‐glucuronidase (GUS) artificial microRNAs (amiRNAs).
