# Supplementary figures and images for: Artificial microRNAs and synthetic trans‐acting small interfering RNAs interfere with viroid infection
Source: Mol Plant Pathol. 2017 Mar 9;18(5):746–53. doi: 10.1111/mpp.12529 (PMC6638287; doi:10.1111/mpp.12529)

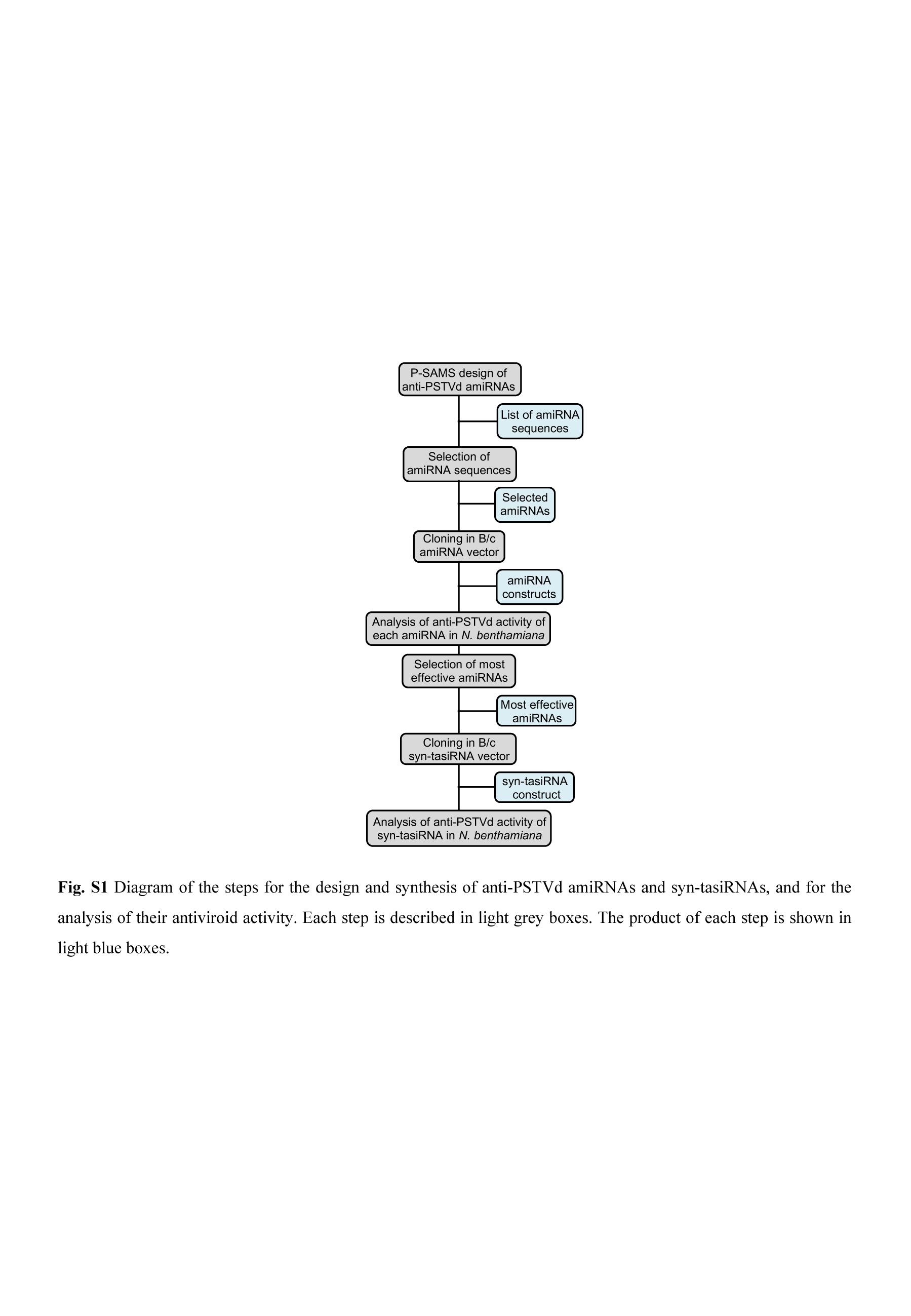

Supplement: Supplementary file 1 — Fig. S1 Diagram of the steps for the design and synthesis of anti‐Potato spindle tuber viroid (PSTVd) artificial microRNAs (amiRNAs) and synthetic trans‐acting small interfering RNAs (syn‐tasiRNAs), and for the analysis of their antiviroid activity. [file MPP-18-746-s001.jpg]

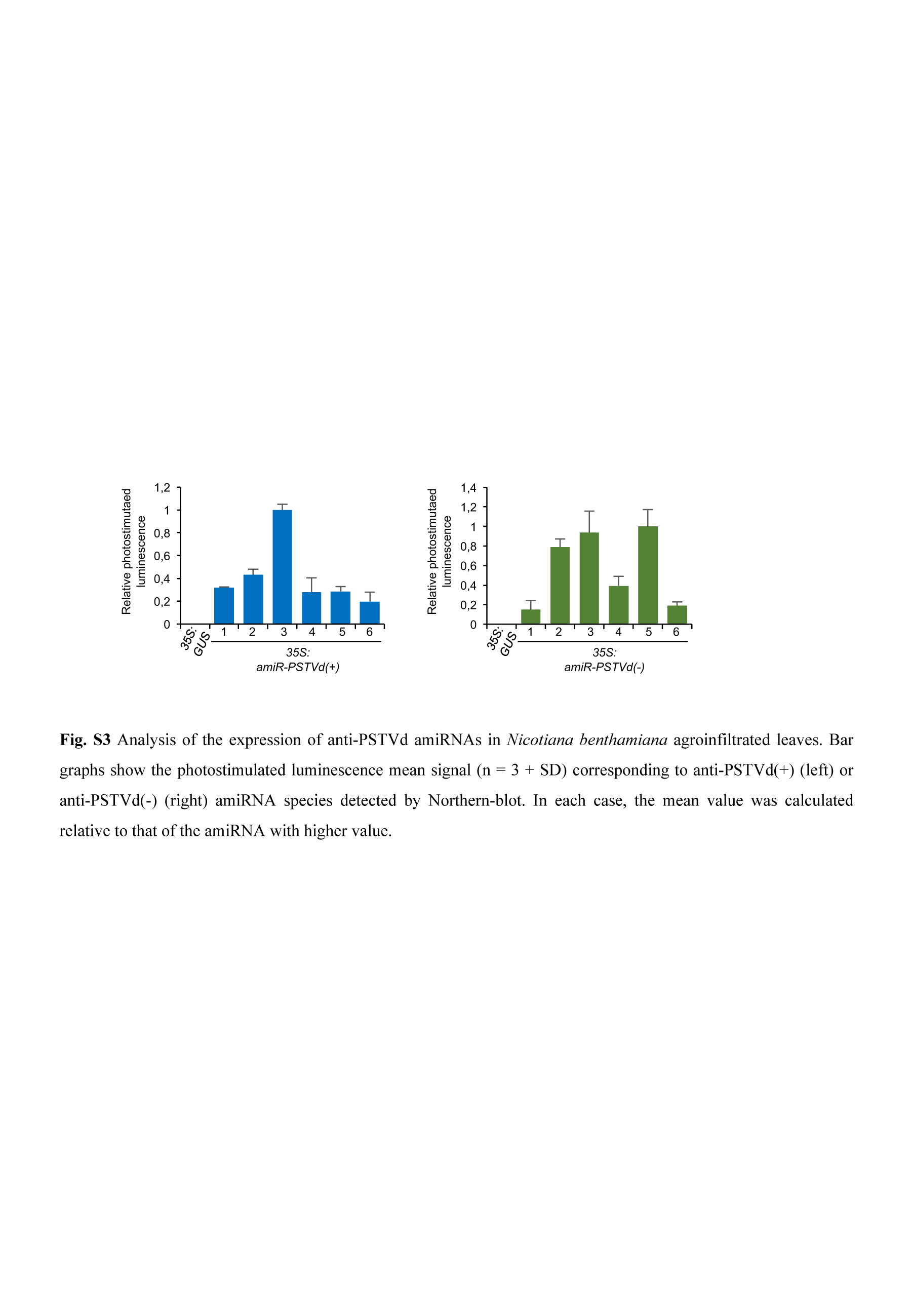

Supplement: Supplementary file 3 — Fig. S3 Analysis of the expression of anti‐Potato spindle tuber viroid (PSTVd) artificial microRNAs (amiRNAs) in Nicotiana benthamiana agroinfiltrated leaves. [file MPP-18-746-s003.jpg]

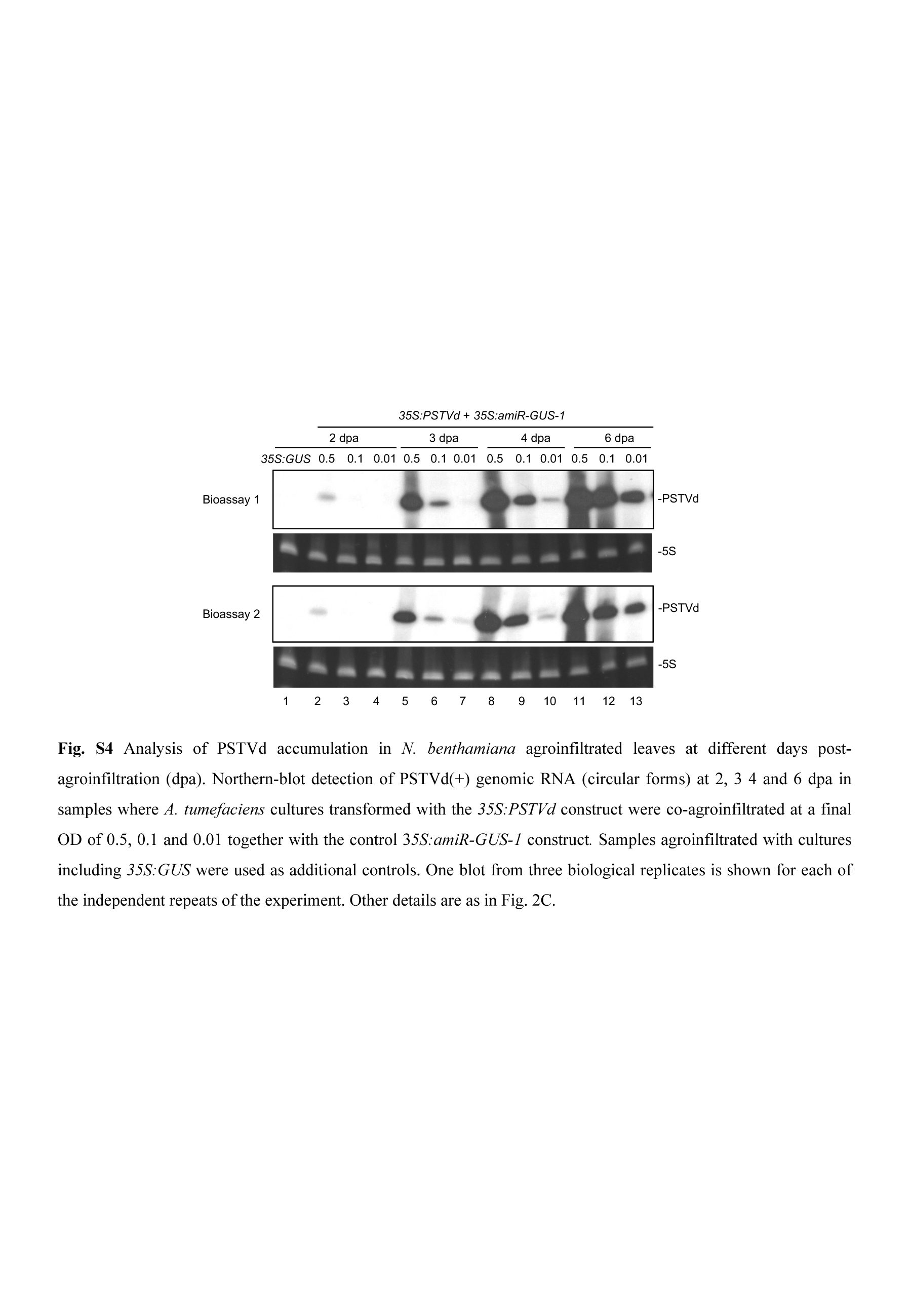

Supplement: Supplementary file 4 — Fig. S4 Analysis of Potato spindle tuber viroid (PSTVd) accumulation in Nicotiana benthamiana agroinfiltrated leaves at different days post‐agroinfiltration (dpa). [file MPP-18-746-s004.jpg]
