# Supplementary material for: Artificial microRNAs and synthetic trans‐acting small interfering RNAs interfere with viroid infection
Source: Mol Plant Pathol. 2017 Mar 9;18(5):746–53. doi: 10.1111/mpp.12529 (PMC6638287; doi:10.1111/mpp.12529)
Supplement: Supplementary file 2 — Fig. S2 Base pairing of artificial microRNAs (amiRNAs) and target β‐glucuronidase (GUS) RNAs. [file MPP-18-746-s002.pdf]

```
amiR-GUS-1   5´UAUUGACCCACACUUUGCCGA 3′
               ||||||||||||||||||||
       GUS   3´AUAACUGGGUGUGAAACGGCA 5′
 (280-300)               ↑     TPS = 1

amiR-GUS-2   5´UAACCUUCACCCGGUUGCCAC 3′
               ||||||||||||||||||||
       GUS   3´AUUGGAAGUGGGCCAACGGUC 5′
 (722-742)               ↑     TPS = 1
```

Fig. S2 Base-pairing of amiRNAs and target GUS RNAs. Other details are as in Fig. 1B.
