# Supplementary material for: Artificial microRNAs and synthetic trans‐acting small interfering RNAs interfere with viroid infection
Source: Mol Plant Pathol. 2017 Mar 9;18(5):746–53. doi: 10.1111/mpp.12529 (PMC6638287; doi:10.1111/mpp.12529)
Supplement: Supplementary file 5 — Methods S1 Experimental procedures. [file MPP-18-746-s005.docx]

**EXPERIMENTAL PROCEDURES**

**Artificial small RNA design**

Because *P-SAMS amiRNA Designer* web tool (<http://p-sams.carringtonlab.org/amirna/designer>) (Fahlgren *et al.*, 2016) outputs a list including only three optimal results, a modified version of *P-SAMS amiRNA Designer* script (<https://github.com/carringtonlab/p-sams>) returning unlimited optimal results was used in order to retrieve the complete list of anti-PSTVd amiRNAs. Due to the circular and/or multimeric nature of PSTVd(+) and PSTVd(-) RNAs, dimeric head-to-tail (1-359;1-359) or tail-to-head (359-1-359-1) sequences of (+) or (-) polarity (Text S2), respectively, corresponding to PSTVd-RG1 (GenBank accession number U23058.1) were used. This strategy assures to retrieve, if existing, optimal results corresponding to amiRNAs targeting sequences flanking nucleotide 1. Full-length sequence of *Escherichia coli* β-glucuronidase gene (GenBank accession number S69414.1) was used for anti-GUS amiRNA design (Text S2). AmiRNA designs were done with the off-targeting filtering in *Solanum lycopersicum* transcriptome iTAGv2.3 (<ftp://ftp.solgenomics.net/tomato_genome/annotation/ITAG2.3_release/>) enabled. *TargetFinder* (Fahlgren et al. 2010) script (<https://github.com/carringtonlab/TargetFinder>) was run to confirm that selected amiRNAs do not target *Nicotiana benthamiana* transcriptome v5.1 (<http://sefapps02.qut.edu.au/benWeb/subpages/downloads.php>) (Table S5) (Nakasugi *et al.*, 2014)

**RNA structure prediction**

The minimal free energy structure predictions of PSTVd monomers of (+) and (-) polarity was determined with *RNAfold* software [ViennaRNA package version 2.2.5, (Lorenz *et al.*, 2011)] using default parameters.

**DNA constructs**

*35S:PSTVd* construct was derived from the binary plasmid pCLEAN-G181 (GenBank accession number EU186083), and included an expression cassette composed by the *Cauliflower mosaic virus* (CaMV) 35S promoter, a modified version of the *Cowpea mosaic virus* (CPMV) RNA-2 5’ untranslated region (UTR) (Sainsbury and Lomonossoff, 2008), a dimeric cDNA (positions G265 to C264) of PSTVd-RG1 (GenBank accession number U23058.1) (Gruner *et al.*, 1995), the CPMV RNA-2 3’ UTR, and the CaMV 35S terminator. *35S:PSTVd* was transformed in an *A. tumefaciens* clone containing the helper plasmid pCLEAN-S48 (Thole *et al.*, 2007).

Artificial sRNA constructs were obtained following the methodology previously described (Carbonell *et al.*, 2014). AmiRNA constructs *35S:amiR-PSTVd(+)-1*, *35S:amiR-PSTVd(+)-2*, *35S:amiR-PSTVd(+)-3, 35S:amiR-PSTVd(+)-4*, *35S:amiR-PSTVd(+)-5*, *35S:amiR-PSTVd(+)-6*, *35S:amiR-PSTVd(-)-1*, *35S:amiR-PSTVd(-)-2*, *35S:amiR-PSTVd(-)-3*, *35S:amiR-PSTVd(-)-4*, *35S:amiR-PSTVd(-)-5*, *35S:amiR-PSTVd(-)-6*, *35S:amiR-GUS-1* and *35S:amiR-GUS-2* were obtained by ligating annealed oligo pairs D2001/D2002, D2003/D2004, D2005/D2006, D2007/D2008, D2009/D2010, D2011/D2012, D2013/D2014, D2015/D2016, D2017/D2018, D2019/D2020, D2021/D2022, and D2023/D2024, respectively (Table S1), into *pMDC32B-AtMIR390a-B/c* (Addgene plasmid #51776) (Carbonell *et al.*, 2014). Syn-tasiRNA constructs *35S:syn-tasiR-PSTVd* and *35S:syn-tasiR-GUS* were obtained by ligating annealed oligo pairs D2165/D2166 and D2167/D2168, respectively (Table S1), into *pMDC32B-AtTAS1c-B/c* (Addgene plasmid #51773) (Carbonell *et al.*, 2014).

*35S:GUS* and *35S:MIR173a* constructs were described before (Montgomery *et al.*, 2008).

**Plant agroinfiltration**

*N. benthamiana* plants were grown in a growth chamber at 25ºC with a 12 h-light/12 h-dark photoperiod. Three week old plants were infiltrated in the third and fourth true leaves with cultures of *A. tumefaciens* GV3101 as described (Cuperus *et al.*, 2010; Llave *et al.*, 2002).

**RNA blot assays**

Total RNA from *N. benthamiana* was isolated in extraction buffer (1M guanidium thiocyanate, 1 M ammonium thiocyanate, 0.1 M sodium acetate pH 5.0, 5% glycerol, 38% water-saturated phenol), followed by one chloroform extraction. RNA was precipitated in 0.5X isopropanol for 20 min. Triplicate samples from pools of infiltrated leaves were analyzed. For viroid genomic RNA and anti-PSTVd amiRNA detection, 15 µg of total RNA was resolved by denaturing PAGE in 5% gels containing 1xTBE and 8 M urea or in 17% gels containing 0.5xTBE and 7M urea, respectively, and transferred to a positively-charged nitrocellulose membrane. Northern-blot hybridizations were done at 70°C or 38ºC in the presence of 50% formamide with strand-specific ^32^P-labeled riboprobes transcribed *in vitro* for genomic viroid RNA or anti-PSTVd amiRNA detection, respectively.

**Sequencing of PSTVd progeny**

cDNA was obtained from a pool of 3 μg of total RNA (1 μg from each biological replicate) using the ReverseAid transcriptase system (Thermo Fisher Scientific). Briefly, for each sample, a mixture including the total RNA and 5 pmol of primer AC4 or AC6 was incubated for 1.5 min at 98 ºC, and then transferred to ice. A 3.5 μl mixture including 2 μl of RT buffer, 1 μl of 10 mM dNTPs, 0.25 μl of RiboLock RNAse Inhibitor (40 U/μL, Thermo Fisher Scientific) and 0.25 μl of ReverseAid transcriptase (200 U/μL, Thermo Fisher Scientific) was added to each sample. Samples were incubated for 45 min at 42 ºC, 10 min at 50 ºC, 5 min at 60 ºC and 15 min at 70 ºC; 0.5 μl of RNase H (5 U/μl, Thermo Fisher Scientific) were added and samples incubated for 30 min at 37 ºC. PCR to amplify PSTVd full-length fragments (359 bp) was done using oligonucleotide pairs AC3/AC4 or AC5/AC6 (Table S5) and Phusion DNA polymerase (Thermo Fisher Scientific) with the following program: 30 sec at 98 ºC; 30 cycles of 98 ºC for 30 sec, 50 ºC for 30 sec, 72 ºC for 30 sec; 10 min at 72 ºC. PCR products were analyzed by 2% agarose gel electrophoresis, and products of the expected size (359 bp) were excised from the gel and sequenced.

**REFERENCES**

**Carbonell, A., Takeda, A., Fahlgren, N., Johnson, S.C., Cuperus, J.T. and Carrington, J.C.** (2014) New generation of artificial microRNA and synthetic *trans*-acting small interfering RNA vectors for efficient gene silencing in Arabidopsis. *Plant Physiol.* **165,** 15-29.

**Cuperus, J.T., Carbonell, A., Fahlgren, N., Garcia-Ruiz, H., Burke, R.T., Takeda, A., Sullivan, C.M., Gilbert, S.D., Montgomery, T.A. and Carrington, J.C.** (2010) Unique functionality of 22-nt miRNAs in triggering RDR6-dependent siRNA biogenesis from target transcripts in Arabidopsis. *Nat. Struct. Mol. Biol.* **17,** 997-U111.

**Fahlgren, N., Hill, S.T., Carrington, J.C. and Carbonell, A.** (2016) P-SAMS: a web site for plant artificial microRNA and synthetic trans-acting small interfering RNA design. *Bioinformatics* **32,** 157-158.

**Gruner, R., Fels, A., Qu, F., Zimmat, R., Steger, G. and Riesner, D.** (1995) Interdependence of pathogenicity and replicability with *Potato spindle tuber viroid*. *Virology* **209,** 60-69.

**Llave, C., Xie, Z., Kasschau, K.D. and Carrington, J.C.** (2002) Cleavage of Scarecrow-like mRNA targets directed by a class of Arabidopsis miRNA. *Science* **297,** 2053-2056.

**Lorenz, R., Bernhart, S.H., Honer Zu Siederdissen, C., Tafer, H., Flamm, C., Stadler, P.F. and Hofacker, I.L.** (2011) ViennaRNA Package 2.0. *Algorithms Mol. Biol.* **6,** 26.

**Montgomery, T.A., Howell, M.D., Cuperus, J.T., Li, D., Hansen, J.E., Alexander, A.L., Chapman, E.J., Fahlgren, N., Allen, E. and Carrington, J.C.** (2008) Specificity of ARGONAUTE7-miR390 interaction and dual functionality in TAS3 *trans*-acting siRNA formation. *Cell* **133,** 128-141.

**Nakasugi, K., Crowhurst, R., Bally, J. and Waterhouse, P.** (2014) Combining transcriptome assemblies from multiple de novo assemblers in the allo-tetraploid plant *Nicotiana benthamiana*. *PLoS One* **9,** e91776.

**Sainsbury, F. and Lomonossoff, G.P.** (2008) Extremely high-level and rapid transient protein production in plants without the use of viral replication. *Plant Physiol.* **148,** 1212-1218.

**Thole, V., Worland, B., Snape, J.W. and Vain, P.** (2007) The pCLEAN dual binary vector system for Agrobacterium-mediated plant transformation. *Plant Physiol.* **145,** 1211-1219.
