# Supplementary material for: Artificial microRNAs and synthetic trans‐acting small interfering RNAs interfere with viroid infection
Source: Mol Plant Pathol. 2017 Mar 9;18(5):746–53. doi: 10.1111/mpp.12529 (PMC6638287; doi:10.1111/mpp.12529)
Supplement: Supplementary file 10 — Table S5 DNA oligonucleotides used in this study. [file MPP-18-746-s010.docx]

**Table S5** DNA oligonucleotides used in this study.

| **Oligonucleotide** | **Sequence** | **Construct/Aim** |
| --- | --- | --- |
| D2001 | TGTATCGGCCGCTGGGCACTCCCCTATGATGATCACATTCGTTATCTATTTTTTAGGGGAGTGCACAGCGGCCGA | *35S:amiR-PSTVd(+)-1* |
| D2002 | AATGTCGGCCGCTGTGCACTCCCCTAAAAAATAGATAACGAATGTGATCATCATAGGGGAGTGCCCAGCGGCCGA |  |
| D2003 | TGTATGCGGGCGCGAGGAAGGACAGATGATGATCACATTCGTTATCTATTTTTTCTGTCCTTCCGCGCGCCCGCA | *35S:amiR-PSTVd(+)-2* |
| D2004 | AATGTGCGGGCGCGCGGAAGGACAGAAAAAATAGATAACGAATGTGATCATCATCTGTCCTTCCTCGCGCCCGCA |  |
| D2005 | TGTATTTCCACCGGGTAGTAGCCGTATGATGATCACATTCGTTATCTATTTTTTACGGCTACTAACCGGTGGAAA | *35S:amiR-PSTVd(+)-3* |
| D2006 | AATGTTTCCACCGGTTAGTAGCCGTAAAAAATAGATAACGAATGTGATCATCATACGGCTACTACCCGGTGGAAA |  |
| D2007 | TGTATTAGTTCCGAGGAACCAACTCATGATGATCACATTCGTTATCTATTTTTTGAGTTGGTTCATCGGAACTAA | *35S:amiR-PSTVd(+)-4* |
| D2008 | AATGTTAGTTCCGATGAACCAACTCAAAAAATAGATAACGAATGTGATCATCATGAGTTGGTTCCTCGGAACTAA |  |
| D2009 | TGTATCCCGGGGATCCCTGAAGCGGATGATGATCACATTCGTTATCTATTTTTTCCGCTTCAGGTATCCCCGGGA | *35S:amiR-PSTVd(+)-5* |
| D2010 | AATGTCCCGGGGATACCTGAAGCGGAAAAAATAGATAACGAATGTGATCATCATCCGCTTCAGGGATCCCCGGGA |  |
| D2011 | TGTATCAAGGGCTAAACACCCTCGGATGATGATCACATTCGTTATCTATTTTTTCCGAGGGTGTGTAGCCCTTGA | *35S:amiR-PSTVd(+)-6* |
| D2012 | AATGTCAAGGGCTACACACCCTCGGAAAAAATAGATAACGAATGTGATCATCATCCGAGGGTGTTTAGCCCTTGA |  |
| D2013 | TGTATGGAACCGCAGTTGGTTCCTGATGATGATCACATTCGTTATCTATTTTTTCAGGAACCAAATGCGGTTCCA | *35S:amiR-PSTVd(-)-1* |
| D2014 | AATGTGGAACCGCATTTGGTTCCTGAAAAAATAGATAACGAATGTGATCATCATCAGGAACCAACTGCGGTTCCA |  |
| D2015 | TGTATGAAGCTCCCGAGAACCGCTAATGATGATCACATTCGTTATCTATTTTTTTAGCGGTTCTAGGGAGCTTCA | *35S:amiR-PSTVd(-)-2* |
| D2016 | AATGTGAAGCTCCCTAGAACCGCTAAAAAAATAGATAACGAATGTGATCATCATTAGCGGTTCTCGGGAGCTTCA |  |
| D2017 | TGTATGTCGCTTCGGCTACTACCCCATGATGATCACATTCGTTATCTATTTTTTGGGGTAGTAGACGAAGCGACA | *35S:amiR-PSTVd(-)-3* |
| D2018 | AATGTGTCGCTTCGTCTACTACCCCAAAAAATAGATAACGAATGTGATCATCATGGGGTAGTAGCCGAAGCGACA |  |
| D2019 | TGTATGTCCTTCCTCGCGCCCGCACATGATGATCACATTCGTTATCTATTTTTTGTGCGGGCGCTAGGAAGGACA | *35S:amiR-PSTVd(-)-4* |
| D2020 | AATGTGTCCTTCCTAGCGCCCGCACAAAAAATAGATAACGAATGTGATCATCATGTGCGGGCGCGAGGAAGGACA |  |
| D2021 | TGTATGGTTCACACCTGACCTCCTCATGATGATCACATTCGTTATCTATTTTTTGAGGAGGTCATGTGTGAACCA | *35S:amiR-PSTVd(-)-5* |
| D2022 | AATGTGGTTCACACATGACCTCCTCAAAAAATAGATAACGAATGTGATCATCATGAGGAGGTCAGGTGTGAACCA |  |
| D2023 | TGTATCCCGGGGAAACCTGGAGCGTATGATGATCACATTCGTTATCTATTTTTTACGCTCCAGGGTTCCCCGGGA | *35S:amiR-PSTVd(-)-6* |
| D2024 | AATGTCCCGGGGAACCCTGGAGCGTAAAAAATAGATAACGAATGTGATCATCATACGCTCCAGGTTTCCCCGGGA |  |
| D2025 | TGTATATTGACCCACACTTTGCCGAATGATGATCACATTCGTTATCTATTTTTTTCGGCAAAGTTTGGGTCAATA | *35S:amiR-GUS-1* |
| D2026 | AATGTATTGACCCAAACTTTGCCGAAAAAAATAGATAACGAATGTGATCATCATTCGGCAAAGTGTGGGTCAATA |  |
| D2027 | TGTATAACCTTCACCCGGTTGCCACATGATGATCACATTCGTTATCTATTTTTTGTGGCAACCGTGTGAAGGTTA | *35S:amiR-GUS-2* |
| D2028 | AATGTAACCTTCACACGGTTGCCACAAAAAATAGATAACGAATGTGATCATCATGTGGCAACCGGGTGAAGGTTA |  |
| D2165 | ATTATGCGGGCGCGAGGAAGGACAGTCCCGGGGATCCCTGAAGCGGTTAGTTCCGAGGAACCAACTCTGGTTCACACCTGACCTCCTCTGTCGCTTCGGCTACTACCCC | *35S:syn-tasiR-PSTVd* |
| D2166 | GTTCGGGGTAGTAGCCGAAGCGACAGAGGAGGTCAGGTGTGAACCAGAGTTGGTTCCTCGGAACTAACCGCTTCAGGGATCCCCGGGACTGTCCTTCCTCGCGCCCGCA |  |
| D2167 | ATTATATTGACCCACACTTTGCCGATAACCTTCACCCGGTTGCCACTATTGACCCACACTTTGCCGATAACCTTCACCCGGTTGCCACTATTGACCCACACTTTGCCGA | *35S:syn-tasiR-GUS* |
| D2168 | GTTCTCGGCAAAGTGTGGGTCAATAGTGGCAACCGGGTGAAGGTTATCGGCAAAGTGTGGGTCAATAGTGGCAACCGGGTGAAGGTTATCGGCAAAGTGTGGGTCAATA |  |
| AC3 | CCGGGGAAACCTGGAGCGAAC | Sequencing of PSTVd progeny |
| AC4 | GGATCCCTGAAGCGCTCCTCC | Sequencing of PSTVd progeny |
| AC5 | CCGAGAACCGCTTTTTCTCTATCTTAC | Sequencing of PSTVd progeny |
| AC6 | GAGCTTCAGTTGTTTCCACCGGGTAG | Sequencing of PSTVd progeny |
