# Supplementary material for: Artificial microRNAs and synthetic trans‐acting small interfering RNAs interfere with viroid infection
Source: Mol Plant Pathol. 2017 Mar 9;18(5):746–53. doi: 10.1111/mpp.12529 (PMC6638287; doi:10.1111/mpp.12529)
Supplement: Supplementary file 11 — Text S1 DNA sequences in FASTA format of all artificial small RNA‐generating precursors used in this study. [file MPP-18-746-s011.docx]

**Text S1**

**(A)**

**>AtMIR390a**

TATAGGGGGGAAAAAAAGGTAGTCATCAGATATATATTTTGGTAAGAAAATATAGAAATGAATAATTTCACGTTTAACGAAGAGGAGATGACGTGTGTTCCTTCGAACCCGAGTTTTGTTCGTCTATAAATAGCACCTTCTCTTCTCCTTCTTCCTCACTTCCATCTTTTTAGCTTCACTATCTCTCTATAATCGGTTTTATCTTTCTCTAAGTCACAACCCAAAAAAACAAAGTAGAGAAGAATCTGTAAAGCTCAGGAGGGATAGCGCCATGATGATCACATTCGTTATCTATTTTTTGGCGCTATCCATCCTGAGTTTCATTGGCTCTTCTTACTACAATGAAAAAGGCCGAGGCAAAACGCCTAAAATCACTTGAGAATCAATTCTTTTTACTGTCCATTTAAGCTATCTTTTATAAACGTGTCTTATTTTCTATCTCTTTTGTTTAAACTAAGAAACTATAGTATTTTGTCTAAAACAAAACATGAAAGAACAGATTAGATCTCATCTTTAGTCTC

**>AtMIR390a-PSTVd(+)-1**

TATAGGGGGGAAAAAAAGGTAGTCATCAGATATATATTTTGGTAAGAAAATATAGAAATGAATAATTTCACGTTTAACGAAGAGGAGATGACGTGTGTTCCTTCGAACCCGAGTTTTGTTCGTCTATAAATAGCACCTTCTCTTCTCCTTCTTCCTCACTTCCATCTTTTTAGCTTCACTATCTCTCTATAATCGGTTTTATCTTTCTCTAAGTCACAACCCAAAAAAACAAAGTAGAGAAGAATCTGTATCGGCCGCTGGGCACTCCCCTATGATGATCACATTCGTTATCTATTTTTTAGGGGAGTGCACAGCGGCCGACATTGGCTCTTCTTACTACAATGAAAAAGGCCGAGGCAAAACGCCTAAAATCACTTGAGAATCAATTCTTTTTACTGTCCATTTAAGCTATCTTTTATAAACGTGTCTTATTTTCTATCTCTTTTGTTTAAACTAAGAAACTATAGTATTTTGTCTAAAACAAAACATGAAAGAACAGATTAGATCTCATCTTTAGTCTC

**>AtMIR390a-PSTVd(+)-2**

TATAGGGGGGAAAAAAAGGTAGTCATCAGATATATATTTTGGTAAGAAAATATAGAAATGAATAATTTCACGTTTAACGAAGAGGAGATGACGTGTGTTCCTTCGAACCCGAGTTTTGTTCGTCTATAAATAGCACCTTCTCTTCTCCTTCTTCCTCACTTCCATCTTTTTAGCTTCACTATCTCTCTATAATCGGTTTTATCTTTCTCTAAGTCACAACCCAAAAAAACAAAGTAGAGAAGAATCTGTATGCGGGCGCGAGGAAGGACAGATGATGATCACATTCGTTATCTATTTTTTCTGTCCTTCCGCGCGCCCGCACATTGGCTCTTCTTACTACAATGAAAAAGGCCGAGGCAAAACGCCTAAAATCACTTGAGAATCAATTCTTTTTACTGTCCATTTAAGCTATCTTTTATAAACGTGTCTTATTTTCTATCTCTTTTGTTTAAACTAAGAAACTATAGTATTTTGTCTAAAACAAAACATGAAAGAACAGATTAGATCTCATCTTTAGTCTC

**>AtMIR390a-PSTVd(+)-3**

TATAGGGGGGAAAAAAAGGTAGTCATCAGATATATATTTTGGTAAGAAAATATAGAAATGAATAATTTCACGTTTAACGAAGAGGAGATGACGTGTGTTCCTTCGAACCCGAGTTTTGTTCGTCTATAAATAGCACCTTCTCTTCTCCTTCTTCCTCACTTCCATCTTTTTAGCTTCACTATCTCTCTATAATCGGTTTTATCTTTCTCTAAGTCACAACCCAAAAAAACAAAGTAGAGAAGAATCTGTATTTCCACCGGGTAGTAGCCGTATGATGATCACATTCGTTATCTATTTTTTACGGCTACTAACCGGTGGAAACATTGGCTCTTCTTACTACAATGAAAAAGGCCGAGGCAAAACGCCTAAAATCACTTGAGAATCAATTCTTTTTACTGTCCATTTAAGCTATCTTTTATAAACGTGTCTTATTTTCTATCTCTTTTGTTTAAACTAAGAAACTATAGTATTTTGTCTAAAACAAAACATGAAAGAACAGATTAGATCTCATCTTTAGTCTC

**>AtMIR390a-PSTVd(+)-4**

TATAGGGGGGAAAAAAAGGTAGTCATCAGATATATATTTTGGTAAGAAAATATAGAAATGAATAATTTCACGTTTAACGAAGAGGAGATGACGTGTGTTCCTTCGAACCCGAGTTTTGTTCGTCTATAAATAGCACCTTCTCTTCTCCTTCTTCCTCACTTCCATCTTTTTAGCTTCACTATCTCTCTATAATCGGTTTTATCTTTCTCTAAGTCACAACCCAAAAAAACAAAGTAGAGAAGAATCTGTATTAGTTCCGAGGAACCAACTCATGATGATCACATTCGTTATCTATTTTTTGAGTTGGTTCATCGGAACTAACATTGGCTCTTCTTACTACAATGAAAAAGGCCGAGGCAAAACGCCTAAAATCACTTGAGAATCAATTCTTTTTACTGTCCATTTAAGCTATCTTTTATAAACGTGTCTTATTTTCTATCTCTTTTGTTTAAACTAAGAAACTATAGTATTTTGTCTAAAACAAAACATGAAAGAACAGATTAGATCTCATCTTTAGTCTC

**>AtMIR390a-PSTVd(+)-5**

TATAGGGGGGAAAAAAAGGTAGTCATCAGATATATATTTTGGTAAGAAAATATAGAAATGAATAATTTCACGTTTAACGAAGAGGAGATGACGTGTGTTCCTTCGAACCCGAGTTTTGTTCGTCTATAAATAGCACCTTCTCTTCTCCTTCTTCCTCACTTCCATCTTTTTAGCTTCACTATCTCTCTATAATCGGTTTTATCTTTCTCTAAGTCACAACCCAAAAAAACAAAGTAGAGAAGAATCTGTATCCCGGGGATCCCTGAAGCGGATGATGATCACATTCGTTATCTATTTTTTCCGCTTCAGGTATCCCCGGGACATTGGCTCTTCTTACTACAATGAAAAAGGCCGAGGCAAAACGCCTAAAATCACTTGAGAATCAATTCTTTTTACTGTCCATTTAAGCTATCTTTTATAAACGTGTCTTATTTTCTATCTCTTTTGTTTAAACTAAGAAACTATAGTATTTTGTCTAAAACAAAACATGAAAGAACAGATTAGATCTCATCTTTAGTCTC

**>AtMIR390a-PSTVd(+)-6**

TATAGGGGGGAAAAAAAGGTAGTCATCAGATATATATTTTGGTAAGAAAATATAGAAATGAATAATTTCACGTTTAACGAAGAGGAGATGACGTGTGTTCCTTCGAACCCGAGTTTTGTTCGTCTATAAATAGCACCTTCTCTTCTCCTTCTTCCTCACTTCCATCTTTTTAGCTTCACTATCTCTCTATAATCGGTTTTATCTTTCTCTAAGTCACAACCCAAAAAAACAAAGTAGAGAAGAATCTGTATCAAGGGCTAAACACCCTCGGATGATGATCACATTCGTTATCTATTTTTTCCGAGGGTGTGTAGCCCTTGACATTGGCTCTTCTTACTACAATGAAAAAGGCCGAGGCAAAACGCCTAAAATCACTTGAGAATCAATTCTTTTTACTGTCCATTTAAGCTATCTTTTATAAACGTGTCTTATTTTCTATCTCTTTTGTTTAAACTAAGAAACTATAGTATTTTGTCTAAAACAAAACATGAAAGAACAGATTAGATCTCATCTTTAGTCTC

**>AtMIR390a-PSTVd(-)-1**

TATAGGGGGGAAAAAAAGGTAGTCATCAGATATATATTTTGGTAAGAAAATATAGAAATGAATAATTTCACGTTTAACGAAGAGGAGATGACGTGTGTTCCTTCGAACCCGAGTTTTGTTCGTCTATAAATAGCACCTTCTCTTCTCCTTCTTCCTCACTTCCATCTTTTTAGCTTCACTATCTCTCTATAATCGGTTTTATCTTTCTCTAAGTCACAACCCAAAAAAACAAAGTAGAGAAGAATCTGTATGGAACCGCAGTTGGTTCCTGATGATGATCACATTCGTTATCTATTTTTTCAGGAACCAAATGCGGTTCCACATTGGCTCTTCTTACTACAATGAAAAAGGCCGAGGCAAAACGCCTAAAATCACTTGAGAATCAATTCTTTTTACTGTCCATTTAAGCTATCTTTTATAAACGTGTCTTATTTTCTATCTCTTTTGTTTAAACTAAGAAACTATAGTATTTTGTCTAAAACAAAACATGAAAGAACAGATTAGATCTCATCTTTAGTCTC

**>AtMIR390a-PSTVd(-)-2**

TATAGGGGGGAAAAAAAGGTAGTCATCAGATATATATTTTGGTAAGAAAATATAGAAATGAATAATTTCACGTTTAACGAAGAGGAGATGACGTGTGTTCCTTCGAACCCGAGTTTTGTTCGTCTATAAATAGCACCTTCTCTTCTCCTTCTTCCTCACTTCCATCTTTTTAGCTTCACTATCTCTCTATAATCGGTTTTATCTTTCTCTAAGTCACAACCCAAAAAAACAAAGTAGAGAAGAATCTGTATGAAGCTCCCGAGAACCGCTAATGATGATCACATTCGTTATCTATTTTTTTAGCGGTTCTAGGGAGCTTCACATTGGCTCTTCTTACTACAATGAAAAAGGCCGAGGCAAAACGCCTAAAATCACTTGAGAATCAATTCTTTTTACTGTCCATTTAAGCTATCTTTTATAAACGTGTCTTATTTTCTATCTCTTTTGTTTAAACTAAGAAACTATAGTATTTTGTCTAAAACAAAACATGAAAGAACAGATTAGATCTCATCTTTAGTCTC

**>AtMIR390a-PSTVd(-)-3**

TATAGGGGGGAAAAAAAGGTAGTCATCAGATATATATTTTGGTAAGAAAATATAGAAATGAATAATTTCACGTTTAACGAAGAGGAGATGACGTGTGTTCCTTCGAACCCGAGTTTTGTTCGTCTATAAATAGCACCTTCTCTTCTCCTTCTTCCTCACTTCCATCTTTTTAGCTTCACTATCTCTCTATAATCGGTTTTATCTTTCTCTAAGTCACAACCCAAAAAAACAAAGTAGAGAAGAATCTGTATGTCGCTTCGGCTACTACCCCATGATGATCACATTCGTTATCTATTTTTTGGGGTAGTAGACGAAGCGACACATTGGCTCTTCTTACTACAATGAAAAAGGCCGAGGCAAAACGCCTAAAATCACTTGAGAATCAATTCTTTTTACTGTCCATTTAAGCTATCTTTTATAAACGTGTCTTATTTTCTATCTCTTTTGTTTAAACTAAGAAACTATAGTATTTTGTCTAAAACAAAACATGAAAGAACAGATTAGATCTCATCTTTAGTCTC

**>AtMIR390a-PSTVd(-)-4**

TATAGGGGGGAAAAAAAGGTAGTCATCAGATATATATTTTGGTAAGAAAATATAGAAATGAATAATTTCACGTTTAACGAAGAGGAGATGACGTGTGTTCCTTCGAACCCGAGTTTTGTTCGTCTATAAATAGCACCTTCTCTTCTCCTTCTTCCTCACTTCCATCTTTTTAGCTTCACTATCTCTCTATAATCGGTTTTATCTTTCTCTAAGTCACAACCCAAAAAAACAAAGTAGAGAAGAATCTGTATGTCCTTCCTCGCGCCCGCACATGATGATCACATTCGTTATCTATTTTTTGTGCGGGCGCTAGGAAGGACACATTGGCTCTTCTTACTACAATGAAAAAGGCCGAGGCAAAACGCCTAAAATCACTTGAGAATCAATTCTTTTTACTGTCCATTTAAGCTATCTTTTATAAACGTGTCTTATTTTCTATCTCTTTTGTTTAAACTAAGAAACTATAGTATTTTGTCTAAAACAAAACATGAAAGAACAGATTAGATCTCATCTTTAGTCTC

**>AtMIR390a-PSTVd(-)-5**

TATAGGGGGGAAAAAAAGGTAGTCATCAGATATATATTTTGGTAAGAAAATATAGAAATGAATAATTTCACGTTTAACGAAGAGGAGATGACGTGTGTTCCTTCGAACCCGAGTTTTGTTCGTCTATAAATAGCACCTTCTCTTCTCCTTCTTCCTCACTTCCATCTTTTTAGCTTCACTATCTCTCTATAATCGGTTTTATCTTTCTCTAAGTCACAACCCAAAAAAACAAAGTAGAGAAGAATCTGTATGGTTCACACCTGACCTCCTCATGATGATCACATTCGTTATCTATTTTTTGAGGAGGTCATGTGTGAACCACATTGGCTCTTCTTACTACAATGAAAAAGGCCGAGGCAAAACGCCTAAAATCACTTGAGAATCAATTCTTTTTACTGTCCATTTAAGCTATCTTTTATAAACGTGTCTTATTTTCTATCTCTTTTGTTTAAACTAAGAAACTATAGTATTTTGTCTAAAACAAAACATGAAAGAACAGATTAGATCTCATCTTTAGTCTC

**>AtMIR390a-PSTVd(-)-6**

TATAGGGGGGAAAAAAAGGTAGTCATCAGATATATATTTTGGTAAGAAAATATAGAAATGAATAATTTCACGTTTAACGAAGAGGAGATGACGTGTGTTCCTTCGAACCCGAGTTTTGTTCGTCTATAAATAGCACCTTCTCTTCTCCTTCTTCCTCACTTCCATCTTTTTAGCTTCACTATCTCTCTATAATCGGTTTTATCTTTCTCTAAGTCACAACCCAAAAAAACAAAGTAGAGAAGAATCTGTATCCCGGGGAAACCTGGAGCGTATGATGATCACATTCGTTATCTATTTTTTACGCTCCAGGGTTCCCCGGGACATTGGCTCTTCTTACTACAATGAAAAAGGCCGAGGCAAAACGCCTAAAATCACTTGAGAATCAATTCTTTTTACTGTCCATTTAAGCTATCTTTTATAAACGTGTCTTATTTTCTATCTCTTTTGTTTAAACTAAGAAACTATAGTATTTTGTCTAAAACAAAACATGAAAGAACAGATTAGATCTCATCTTTAGTCTC

**>AtMIR390a-GUS-1**

TATAGGGGGGAAAAAAAGGTAGTCATCAGATATATATTTTGGTAAGAAAATATAGAAATGAATAATTTCACGTTTAACGAAGAGGAGATGACGTGTGTTCCTTCGAACCCGAGTTTTGTTCGTCTATAAATAGCACCTTCTCTTCTCCTTCTTCCTCACTTCCATCTTTTTAGCTTCACTATCTCTCTATAATCGGTTTTATCTTTCTCTAAGTCACAACCCAAAAAAACAAAGTAGAGAAGAATCTGTATATTGACCCACACTTTGCCGAATGATGATCACATTCGTTATCTATTTTTTTCGGCAAAGTTTGGGTCAATACATTGGCTCTTCTTACTACAATGAAAAAGGCCGAGGCAAAACGCCTAAAATCACTTGAGAATCAATTCTTTTTACTGTCCATTTAAGCTATCTTTTATAAACGTGTCTTATTTTCTATCTCTTTTGTTTAAACTAAGAAACTATAGTATTTTGTCTAAAACAAAACATGAAAGAACAGATTAGATCTCATCTTTAGTCTC

**>AtMIR390a-GUS-2**

TATAGGGGGGAAAAAAAGGTAGTCATCAGATATATATTTTGGTAAGAAAATATAGAAATGAATAATTTCACGTTTAACGAAGAGGAGATGACGTGTGTTCCTTCGAACCCGAGTTTTGTTCGTCTATAAATAGCACCTTCTCTTCTCCTTCTTCCTCACTTCCATCTTTTTAGCTTCACTATCTCTCTATAATCGGTTTTATCTTTCTCTAAGTCACAACCCAAAAAAACAAAGTAGAGAAGAATCTGTATAACCTTCACCCGGTTGCCACATGATGATCACATTCGTTATCTATTTTTTGTGGCAACCGTGTGAAGGTTACATTGGCTCTTCTTACTACAATGAAAAAGGCCGAGGCAAAACGCCTAAAATCACTTGAGAATCAATTCTTTTTACTGTCCATTTAAGCTATCTTTTATAAACGTGTCTTATTTTCTATCTCTTTTGTTTAAACTAAGAAACTATAGTATTTTGTCTAAAACAAAACATGAAAGAACAGATTAGATCTCATCTTTAGTCTC

**(B)**

**>AtTAS1c**

AAACCTAAACCTAAACGGCTAAGCCCGACGTCAAATACCAAAAAGAGAAAAACAAGAGCGCCGTCAAGCTCTGCAAATACGATCTGTAAGTCCATCTTAACACAAAAGTGAGATGGGTTCTTAGATCATGTTCCGCCGTTAGATCGAGTCATGGTCTTGTCTCATAGAAAGGTACTTTCGTTTACTTCTTTTGAGTATCGAGTAGAGCGTCGTCTATAGTTAGTTTGAGATTGCGTTTGTCAGAAGTTAGGTTCAATGTCCCGGTCCAATTTTCACCAGCCATGTGTCAGTTTCGTTCCTTCCCGTCCTCTTCTTTGATTTCGTTGGGTTACGGATGTTTTCGAGATGAAACAGCATTGTTTTGTTGTGATTTTTCTCTACAAGCGAATAGACCATTTATCGGTGGATCTTAGAAAATTATTCTAAGTCCAACATAGCGTATTCTAAGTTCAACATATCGACGAACTAGAAAAGACATTGGACATATTCCAGGATATGCAAAAGAAAACAATGAATATTGTTTTGAATGTGTTCAAGTAAATGAGATTTTCAAGTCGTCTAAAGAACAGTTGCTAATACAGTTACTTATTTCAATAAATAATTGGTTCTAATAATACAAAACATATTCGAGGATATGCAGAAAAAAAGATGTTTGTTATTTTGAAAAGCTTGAGTAGTTTCTCTCCGAGGTGTAGCGAAGAAGCATCATCTACTTTGTAATGTAATTTTCTTTATGTTTTCACTTTGTAATTTTATTTGTGTTAATGTACCATGGCCGATATCGGTTTTATTGAAAGAAAATTTATGTTACTTCTGTTTTGGCTTTGCAATCAGTTATGCTAGTTTTCTTATACCCTTTCGTAAGCTTCCTAAGGAATCGTTCATTGATTTCCACTGCTTCATTGTATATTAAAACTTTACAACTGTATCGACCATCATATAATTCTGGGTCAAGAGATGAAAATAGAACACCACATCGTAAAGTGAAAT

**>AtTAS1c-PSTVd**

AAACCTAAACCTAAACGGCTAAGCCCGACGTCAAATACCAAAAAGAGAAAAACAAGAGCGCCGTCAAGCTCTGCAAATACGATCTGTAAGTCCATCTTAACACAAAAGTGAGATGGGTTCTTAGATCATGTTCCGCCGTTAGATCGAGTCATGGTCTTGTCTCATAGAAAGGTACTTTCGTTTACTTCTTTTGAGTATCGAGTAGAGCGTCGTCTATAGTTAGTTTGAGATTGCGTTTGTCAGAAGTTAGGTTCAATGTCCCGGTCCAATTTTCACCAGCCATGTGTCAGTTTCGTTCCTTCCCGTCCTCTTCTTTGATTTCGTTGGGTTACGGATGTTTTCGAGATGAAACAGCATTGTTTTGTTGTGATTTTTCTCTACAAGCGAATAGACCATTTATCGGTGGATCTTAGAAAATTATGCGGGCGCGAGGAAGGACAGTCCCGGGGATCCCTGAAGCGGTTAGTTCCGAGGAACCAACTCTGGTTCACACCTGACCTCCTCTGTCGCTTCGGCTACTACCCCGAACTAGAAAAGACATTGGACATATTCCAGGATATGCAAAAGAAAACAATGAATATTGTTTTGAATGTGTTCAAGTAAATGAGATTTTCAAGTCGTCTAAAGAACAGTTGCTAATACAGTTACTTATTTCAATAAATAATTGGTTCTAATAATACAAAACATATTCGAGGATATGCAGAAAAAAAGATGTTTGTTATTTTGAAAAGCTTGAGTAGTTTCTCTCCGAGGTGTAGCGAAGAAGCATCATCTACTTTGTAATGTAATTTTCTTTATGTTTTCACTTTGTAATTTTATTTGTGTTAATGTACCATGGCCGATATCGGTTTTATTGAAAGAAAATTTATGTTACTTCTGTTTTGGCTTTGCAATCAGTTATGCTAGTTTTCTTATACCCTTTCGTAAGCTTCCTAAGGAATCGTTCATTGATTTCCACTGCTTCATTGTATATTAAAACTTTACAACTGTATCGACCATCATATAATTCTGGGTCAAGAGATGAAAATAGAACACCACATCGTAAAGTGAAAT

**>AtTAS1c-GUS**

AAACCTAAACCTAAACGGCTAAGCCCGACGTCAAATACCAAAAAGAGAAAAACAAGAGCGCCGTCAAGCTCTGCAAATACGATCTGTAAGTCCATCTTAACACAAAAGTGAGATGGGTTCTTAGATCATGTTCCGCCGTTAGATCGAGTCATGGTCTTGTCTCATAGAAAGGTACTTTCGTTTACTTCTTTTGAGTATCGAGTAGAGCGTCGTCTATAGTTAGTTTGAGATTGCGTTTGTCAGAAGTTAGGTTCAATGTCCCGGTCCAATTTTCACCAGCCATGTGTCAGTTTCGTTCCTTCCCGTCCTCTTCTTTGATTTCGTTGGGTTACGGATGTTTTCGAGATGAAACAGCATTGTTTTGTTGTGATTTTTCTCTACAAGCGAATAGACCATTTATCGGTGGATCTTAGAAAATTATATTGACCCACACTTTGCCGATAACCTTCACCCGGTTGCCACTATTGACCCACACTTTGCCGATAACCTTCACCCGGTTGCCACTATTGACCCACACTTTGCCGAGAACTAGAAAAGACATTGGACATATTCCAGGATATGCAAAAGAAAACAATGAATATTGTTTTGAATGTGTTCAAGTAAATGAGATTTTCAAGTCGTCTAAAGAACAGTTGCTAATACAGTTACTTATTTCAATAAATAATTGGTTCTAATAATACAAAACATATTCGAGGATATGCAGAAAAAAAGATGTTTGTTATTTTGAAAAGCTTGAGTAGTTTCTCTCCGAGGTGTAGCGAAGAAGCATCATCTACTTTGTAATGTAATTTTCTTTATGTTTTCACTTTGTAATTTTATTTGTGTTAATGTACCATGGCCGATATCGGTTTTATTGAAAGAAAATTTATGTTACTTCTGTTTTGGCTTTGCAATCAGTTATGCTAGTTTTCTTATACCCTTTCGTAAGCTTCCTAAGGAATCGTTCATTGATTTCCACTGCTTCATTGTATATTAAAACTTTACAACTGTATCGACCATCATATAATTCTGGGTCAAGAGATGAAAATAGAACACCACATCGTAAAGTGAAAT

**Text S1** DNA sequence in FASTA format of all artificial small RNA generating precursors used in this study. (A) *AtMIR390a*-based amiRNA foldbacks. Sequence unique to the pri-*AtMIR390a*, pre-*AtMIR390a*, miRNA/amiRNA guide strand and miRNA*/amiRNA* strand sequences are highlighted in black, white, blue and green, respectively. Nucleotides of the pre-*AtMIR390a* that had to be modified to preserve the authentic *AtMIR390a* precursor structure are highlighted in red. (B) Sequence corresponding to miR173 target site is highlighted in blue. Sequence corresponding to tasiRNA-2 (position 3´D2[+]), tasiRNA-3 (position 3´D3[+]) and tasiRNA-4 (position 3´D4[+]) is highlighted in blue, green, dark and light pink, respectively. Sequence corresponding to the syn-tasiRNA cassete is highlighted in yellow. All the other sequences from Arabidopsis *TAS1c* gene are highlighted in black.
