# Supplementary material for: Artificial microRNAs and synthetic trans‐acting small interfering RNAs interfere with viroid infection
Source: Mol Plant Pathol. 2017 Mar 9;18(5):746–53. doi: 10.1111/mpp.12529 (PMC6638287; doi:10.1111/mpp.12529)
Supplement: Supplementary file 12 — Text S2 DNA sequences in FASTA format used in P‐SAMS amiRNA Designer to design anti‐Potato spindle tuber viroid (PSTVd) or anti‐β‐glucuronidase (GUS) artificial microRNAs (amiRNAs). [file MPP-18-746-s012.docx]

**Text S2**

>dPSTVd(+) (1-359;1-359)

CGGAACTAAACTCGTGGTTCCTGTGGTTCACACCTGACCTCCTGACAAGAAAAGAAAAAAGAAGGCGGCTCGGAGGAGCGCTTCAGGGATCCCCGGGGAAACCTGGAGCGAACTGGCAAAAAAGGACGGTGGGGAGTGCCCAGCGGCCGACAGGAGTAATTCCCGCCGAAACAGGGTTTTCACCCTTCCTTTCTTCGGGTGTCCTTCCTCGCGCCCGCAGGACCACCCCTCGCCCCCTTTGCGCTGTCGCTTCGGCTACTACCCGGTGGAAACAACTGAAGCTCCCGAGAACCGCTTTTTCTCTATCTTACTTGCTCCGGGGCGAGGGTGTTTAGCCCTTGGAACCGCAGTTGGTTCCTCGGAACTAAACTCGTGGTTCCTGTGGTTCACACCTGACCTCCTGACAAGAAAAGAAAAAAGAAGGCGGCTCGGAGGAGCGCTTCAGGGATCCCCGGGGAAACCTGGAGCGAACTGGCAAAAAAGGACGGTGGGGAGTGCCCAGCGGCCGACAGGAGTAATTCCCGCCGAAACAGGGTTTTCACCCTTCCTTTCTTCGGGTGTCCTTCCTCGCGCCCGCAGGACCACCCCTCGCCCCCTTTGCGCTGTCGCTTCGGCTACTACCCGGTGGAAACAACTGAAGCTCCCGAGAACCGCTTTTTCTCTATCTTACTTGCTCCGGGGCGAGGGTGTTTAGCCCTTGGAACCGCAGTTGGTTCCT

>dPSTVd(-) (359-1; 359-1)

AGGAACCAACTGCGGTTCCAAGGGCTAAACACCCTCGCCCCGGAGCAAGTAAGATAGAGAAAAAGCGGTTCTCGGGAGCTTCAGTTGTTTCCACCGGGTAGTAGCCGAAGCGACAGCGCAAAGGGGGCGAGGGGTGGTCCTGCGGGCGCGAGGAAGGACACCCGAAGAAAGGAAGGGTGAAAACCCTGTTTCGGCGGGAATTACTCCTGTCGGCCGCTGGGCACTCCCCACCGTCCTTTTTTGCCAGTTCGCTCCAGGTTTCCCCGGGGATCCCTGAAGCGCTCCTCCGAGCCGCCTTCTTTTTTCTTTTCTTGTCAGGAGGTCAGGTGTGAACCACAGGAACCACGAGTTTAGTTCCGAGGAACCAACTGCGGTTCCAAGGGCTAAACACCCTCGCCCCGGAGCAAGTAAGATAGAGAAAAAGCGGTTCTCGGGAGCTTCAGTTGTTTCCACCGGGTAGTAGCCGAAGCGACAGCGCAAAGGGGGCGAGGGGTGGTCCTGCGGGCGCGAGGAAGGACACCCGAAGAAAGGAAGGGTGAAAACCCTGTTTCGGCGGGAATTACTCCTGTCGGCCGCTGGGCACTCCCCACCGTCCTTTTTTGCCAGTTCGCTCCAGGTTTCCCCGGGGATCCCTGAAGCGCTCCTCCGAGCCGCCTTCTTTTTTCTTTTCTTGTCAGGAGGTCAGGTGTGAACCACAGGAACCACGAGTTTAGTTCCG

>GUS (1-1812)

ATGGTCCGTCCTGTAGAAACCCCAACCCGTGAAATCAAAAAACTCGACGGCCTGTGGGCATTCAGTCTGGATCGCGAAAACTGTGGAATTGATCAGCGTTGGTGGGAAAGCGCGTTACAAGAAAGCCGGGCAATTGCTGTGCCAGGCAGTTTTAACGATCAGTTCGCCGATGCAGATATTCGTAATTATGCGGGCAACGTCTGGTATCAGCGCGAAGTCTTTATACCGAAAGGTTGGGCAGGCCAGCGTATCGTGCTGCGTTTCGATGCGGTCACTCATTACGGCAAAGTGTGGGTCAATAATCAGGAAGTGATGGAGCATCAGGGCGGCTATACGCCATTTGAAGCCGATGTCACGCCGTATGTTATTGCCGGGAAAAGTGTACGTATCACCGTTTGTGTGAACAACGAACTGAACTGGCAGACTATCCCGCCGGGAATGGTGATTACCGACGAAAACGGCAAGAAAAAGCAGTCTTACTTCCATGATTTCTTTAACTATGCCGGAATCCATCGCAGCGTAATGCTCTACACCACGCCGAACACCTGGGTGGACGATATCACCGTGGTGACGCATGTCGCGCAAGACTGTAACCACGCGTCTGTTGACTGGCAGGTGGTGGCCAATGGTGATGTCAGCGTTGAACTGCGTGATGCGGATCAACAGGTGGTTGCAACTGGACAAGGCACTAGCGGGACTTTGCAAGTGGTGAATCCGCACCTCTGGCAACCGGGTGAAGGTTATCTCTATGAACTGTGCGTCACAGCCAAAAGCCAGACAGAGTGTGATATCTACCCGCTTCGCGTCGGCATCCGGTCAGTGGCAGTGAAGGGCGAACAGTTCCTGATTAACCACAAACCGTTCTACTTTACTGGCTTTGGTCGTCATGAAGATGCGGACTTACGTGGCAAAGGATTCGATAACGTGCTGATGGTGCACGACCACGCATTAATGGACTGGATTGGGGCCAACTCCTACCGTACCTCGCATTACCCTTACGCTGAAGAGATGCTCGACTGGGCAGATGAACATGGCATCGTGGTGATTGATGAAACTGCTGCTGTCGGCTTTAACCTCTCTTTAGGCATTGGTTTCGAAGCGGGCAACAAGCCGAAAGAACTGTACAGCGAAGAGGCAGTCAACGGGGAAACTCAGCAAGCGCACTTACAGGCGATTAAAGAGCTGATAGCGCGTGACAAAAACCACCCAAGCGTGGTGATGTGGAGTATTGCCAACGAACCGGATACCCGTCCGCAAGGTGCACGGGAATATTTCGCGCCACTGGCGGAAGCAACGCGTAAACTCGACCCGACGCGTCCGATCACCTGCGTCAATGTAATGTTCTGCGACGCTCACACCGATACCATCAGCGATCTCTTTGATGTGCTGTGCCTGAACCGTTATTACGGATGGTATGTCCAAAGCGGCGATTTGGAAACGGCAGAGAAGGTACTGGAAAAAGAACTTCTGGCCTGGCAGGAGAAACTGCATCAGCCGATTATCATCACCGAATACGGCGTGGATACGTTAGCCGGGCTGCACTCAATGTACACCGACATGTGGAGTGAAGAGTATCAGTGTGCATGGCTGGATATGTATCACCGCGTCTTTGATCGCGTCAGCGCCGTCGTCGGTGAACAGGTATGGAATTTCGCCGATTTTGCGACCTCGCAAGGCATATTGCGCGTTGGCGGTAACAAGAAAGGGATCTTCACTCGCGACCGCAAACCGAAGTCGGCGGCTTTTCTGCTGCAAAAACGCTGGACTGGCATGAACTTCGGTGAAAAACCGCAGCAGGGAGGCAAACAATAA

**Text S2** DNA sequence in FASTA format used in P-SAMS amiRNA Designer to design anti-PSTVd or anti-GUS amiRNAs.
